# Global Sensitivity Analysis of Ventricular Myocyte Model-Derived Metrics for Proarrhythmic Risk Assessment

**DOI:** 10.3389/fphar.2019.01054

**Published:** 2019-10-02

**Authors:** Jaimit Parikh, Paolo Di Achille, James Kozloski, Viatcheslav Gurev

**Affiliations:** IBM T.J. Watson Research Center, Yorktown, NY, United States

**Keywords:** global sensitivity analysis, torsades de pointes, computational modeling, early afterdepolarizations, ion channel pharmacology

## Abstract

Multiscale computational models of the heart are being extensively investigated for improved assessment of drug-induced torsades de pointes (TdP) risk, a fatal side effect of many drugs. Model-derived metrics such as action potential duration and net charge carried by ionic currents (*qNet*) have been proposed as potential candidates for TdP risk stratification after being tested on small datasets. Unlike purely statistical approaches, model-derived metrics are thought to provide mechanism-based classification. In particular, *qNet* has been recently proposed as a surrogate metric for early afterdepolarizations (EADs), which are known to be cellular triggers of TdP. Analysis of critical model components and of the ion channels that have major impact on model-derived metrics can lead to improvements in the confidence of the prediction. In this paper, we analyze large populations of virtual drugs to systematically examine the influence of different ion channels on model-derived metrics that have been proposed for proarrhythmic risk assessment. We demonstrate *via* global sensitivity analysis (GSA) that model-derived metrics are most sensitive to different sets of input parameters. Similarly, important differences in sensitivity to specific channel blocks are highlighted when classifying drugs into different risk categories by either *qNet* or a metric directly based on simulated EADs. In particular, the higher sensitivity of *qNet* to the block of the late sodium channel might explain why its classification accuracy is better than that of the EAD-based metric, as shown for a small set of known drugs. Our results highlight the need for a better mechanistic interpretation of promising metrics like *qNet* based on a formal analysis of models. GSA should, therefore, constitute an essential component of the *in silico* workflow for proarrhythmic risk assessment, as an improved understanding of the structure of model-derived metrics could increase confidence in model-predicted risk.

## Introduction

Drug-induced torsades de pointes (TdP) is a specific form of polymorphic ventricular tachycardia that may lead to ventricular fibrillation and sudden cardiac death (Yap and Camm, 2003). Several drugs have been withdrawn from the market in the past due to TdP risk Gintant, (2008). Although the current clinical safety guidelines are successfully preventing drugs with torsadogenic risk from reaching the market (Sager et al., 2014), safe drugs may be potentially excluded due to the low specificity of the screening process, which targets only the hERG channels. The Comprehensive *in vitro* Proarrhythmia Assay (CiPA) is a global initiative to provide revised guidelines for better evaluation of the proarrhythmic risk of drugs (Fermini et al., 2016). *In silico* evaluation of proarrhythmic action for different compounds constitutes an important foundation under the CiPA initiative to link data from *in vitro* assays to changes in cell behavior (Colatsky et al., 2016; Fermini et al., 2016).

The main component of the *in silico* evaluation are classifiers that are based on the so-called “derived features,” input variables for the classifiers that are extracted from the outputs of biophysical models. The term “direct features” refers instead to the original feature set estimated from experiments investigating how drugs affect ion channel kinetics. Biophysical models serve as complex transformations that generate feature sets conditioned to the prior knowledge used in creating the model, thus potentially improving the efficacy of linear classifiers in inferring TdP risk. Diverse sets of derived features have been suggested as potential candidates for TdP risk classification (**Table 1**). In one of the earliest works on the use of the myocyte models for TdP risk prediction, simulated action potential duration at 90% repolarization (*APD*90) was shown to provide the best discrimination between torsadogenic and non-torsadogenic drugs (Mirams et al., 2011). Other derived features extracted from the action potential [e.g., early afterdepolarization (EAD) and transmural dispersion of repolarization (TDR)] have also been suggested as possible candidate metrics for TdP risk prediction (Christophe, 2013, Christophe, 2015). Considering derived features from calcium transient in addition to features of the action potential has been shown to improve TdP risk discrimination (Lancaster and Sobie, 2016). Recently, tertiary TdP risk classifiers trained on a set of 12 drugs categorized into three clinical TdP risk groups (high, intermediate, and low/no risk) have been developed at FDA as part of the CiPA initiative (Dutta et al., 2017; Li et al., 2017). Finally, two new derived features *cqInward* (Li et al., 2017) and *qNet* (Dutta et al., 2017) have been proposed to separate the 12 training drugs into desired target groups. The *qNet* metric was further validated on 16 test compounds (Li et al., 2018). Uncertainty quantification methods (Johnstone et al., 2016) have recently gained increased attention due to their ability to better estimate the confidence of the model-predicted risk (Chang et al., 2017) by taking into account noise in the *in vitro* measurements of drug-induced effects on ionic currents, under the CiPA initiative.

**Table 1:** Previously proposed derived features.

Feature	*In silico* model	# Compounds tested	Reference
*APD_90_*	Ventricular myocyte models of rabbit, rat and human	31	Mirams et al. (2011)
*C_drug,EAD_/EFTPC*	Human ventricular myocyte model	31 from Mirams et al. (2011)	Christophe (2013)
*TDR*	Human ventricular myocyte model	55 from Kramer et al. (2013)	Christophe (2015)
*C_drug,Arrhythmia_/EFTPC*	3D FEM model of human heart	12	Okada et al. (2015)
*APD_50_ & DiastolicCa^2+^*	Human ventricular myocyte model	86 from Mirams et al. (2011); Kramer et al. (2013)	Lancaster and Sobie (2016)
*cqInward*	Human ventricular myocyte model	12	Li et al. (2017)
*TdP_population,score_*	Human ventricular myocyte model	62 (55 from Kramer et al. (2013))	Passini et al. (2017)
*qNet*	Human ventricular myocyte model	12	Dutta et al. (2017)

C_drug,EAD_ - concentration of the drug that produces EAD.

C_drug,Arrhythmia_ - concentration of the drug that produces arrhythmia in the model.

TDR - Transmural dispersion.

cqInward - metric that quantifies the change in the amount of charge carried by INaL and ICaL.

APD90 - Action potential duration at 90% amplitude,

APD50 - Action potential duration at 50% amplitude,

DiastolicCa^2+^ - Diastolic calcium concentration, and

TdP_population,score_ - The fraction of models developing repolarization abnormalities (RA) multiplied by a factor inversely related to the drug concentration at which those RA occur.

Model-derived features that are directly linked to drug-induced changes in myocyte membrane activity are promising metrics to provide mechanism-based classification of compounds into different risk categories. A simple model-derived feature, *qNet* (Dutta et al., 2017), has recently been shown to provide good risk discrimination and was proposed as a surrogate for the propensity to EADs, which are known triggers of TdP (Yan et al., 2001). In this paper, we apply global sensitivity analysis (GSA) to the existing CiPA *in silico* framework to identify the key model components that derived metrics are most sensitive to. We also identify the inputs that are important for classifying virtual drugs into different risk groups based either on an EAD metric or on *qNet*. In agreement with a recent report (McMillan et al., 2017) showing better performance of classifiers built on simple metrics such as *APD*90, we find that *qNet* performs better than the EAD metric in classifying torsadogenic risk. Our results indicate that, despite being well correlated to metrics directly based on EADs, *qNet* also depends on additional parameters that seem to confer its better performance. Hence, our results highlight the need for a better mechanistic understanding of promising model-derived metrics. In addition, our sensitivity analysis provides an explanation for the similar risk classification performances achieved by direct and derived features.

## Methods

The CiPAORd Model and Input Parameters section describes the *in silico* model used in the paper. To perform GSA, we generated large sets of virtual drugs, i.e., sets of perturbations to the ion channels parameters of the model. The details of the input parameters considered for generating the virtual drug population are presented in Sampling Virtual Drug Populations section. Responses to the virtual drugs were examined, and several model-derived features such as *APD*_90_, *qNet*, and peak calcium concentration (*peakCa*) were estimated. The section *In Silico* Simulations and Derived Features presents details on the derived features extracted from the *in silico* model. Virtual drugs were also tested for their ability to induce EADs. In the section EAD protocol we discuss the protocol used to test for EAD generation in the model. The methods used for GSA are described in the GSA section. Finally, the methods for classifying the 28 drugs selected under the CiPA initiative, which we refer to as “CiPA drugs,” with respect to their defined torsadogenic risk are described in the section Tertiary Risk Stratification of “CiPA Drugs.”

### CiPAORd Model and Input Parameters

**Figure 1:**
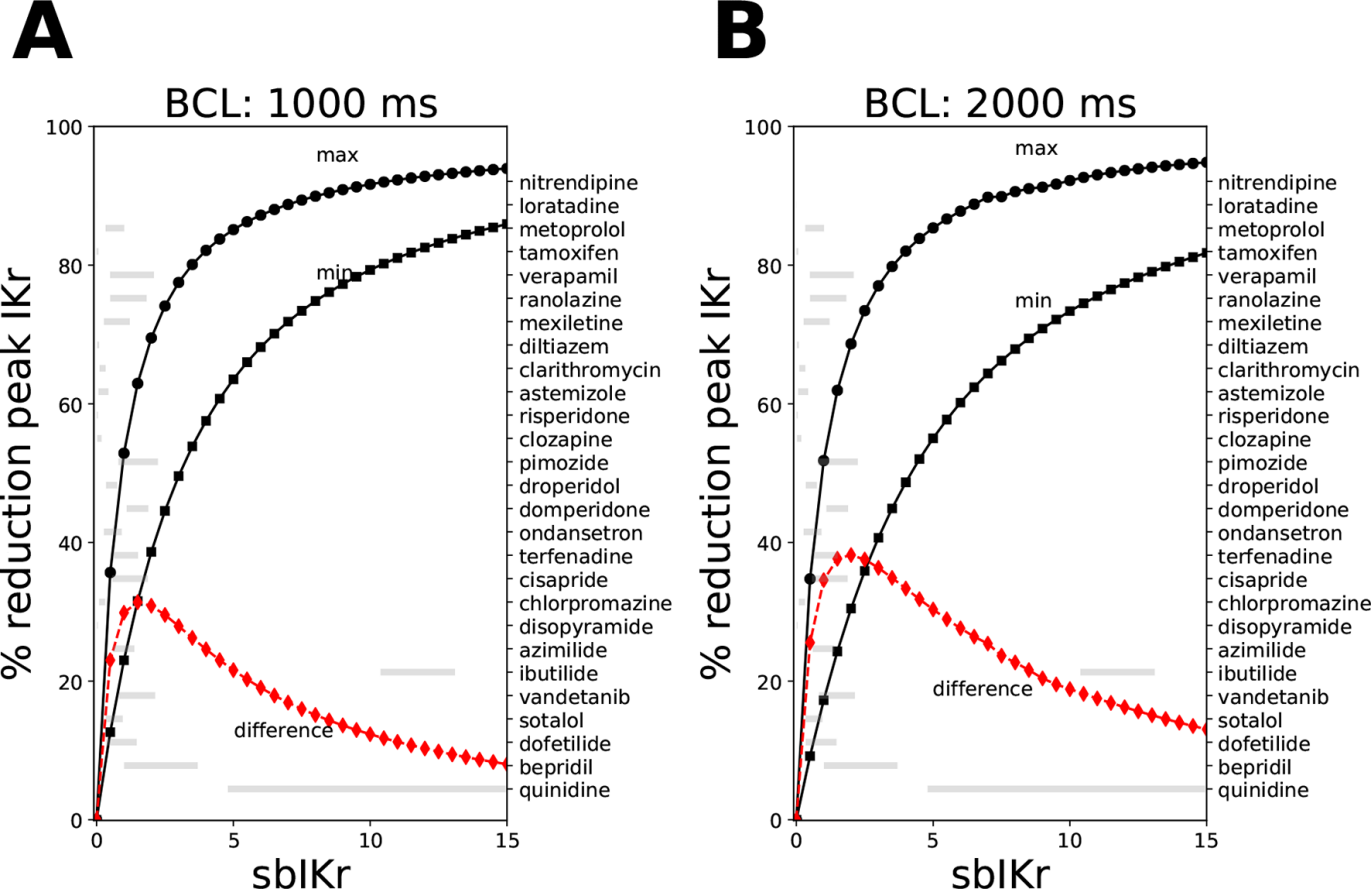
Reduction in peak *IKr* current for the CiPAORd model at a fixed value of the *sbIKr* parameter while allowing the dynamic parameters *Ku* and *Vhalf* to vary across the ranges 1e−5 to 1, and −200 to −1, respectively. Changes in peak *IKr* current after 1,000 beats of simulation at **(A)** a basic cycle length of 1,000 ms and **(B)** a basic cycle length of 2,000 ms. The solid line with square markers shows the minimum reduction in peak *IKr* current obtained at parameter values of 1e−5 for *Ku* and −200 for *Vhalf*. The maximum reduction in peak *IKr* current was plotted as a solid line with circular markers. The red line indicates the difference between the maximum and the minimum extremes. The variations in *sbIKr* parameter for each of the 28 “CiPA drugs” at 1–4× EFTPC values is also shown as gray bars.

In this study, we perform GSA on the CiPAORdv1.0 endo-cell model type, i.e., the optimized model from Dutta et al. (2017). The CiPAORd model was developed at the FDA by modifying the O’Hara-Rudy ventricular myocyte model (O’Hara et al., 2011) to include dynamic drug-hERG interactions for improved proarrhythmic risk assessment (Li et al., 2017). To simulate virtual drug effects, we varied nine input parameters (**Table 2**). Metric sensitivity to hERG current was evaluated by modulating three parameters (Li et al., 2017): *E_max_*, which describes the concentration response of the drug; *Ku*, which indicates the unbinding reaction rate; and *Vhalf*, which represents the membrane voltage at which half of the drug-bound channels are open. In this paper, we refer to the *E_max_* parameter that represents the static component of the hERG block as *sbIKr*, which is given by

(1) $$\mathrm{sbIKr}=K_{\max}\frac{C_{\mathrm{drug}}^{h}}{IC_{50}^{h}+C_{\mathrm{drug}}^{h}},$$

where *C_drug_* is the drug concentration, *K_max_* is the maximum drug effect at saturating concentrations, *h* is the Hill coefficient, and *IC*_50_ is the concentration where half maximum effect is achieved. **Figure 1** shows the relationship between the *sbIKr* parameter and the peak *IKr* current for two pacing rates, 1,000 ms (panel A) and 2,000 ms (panel B). The minimum reduction in peak *IKr* current was obtained for *Ku* = 1e−5 and *Vhalf* = −200 (see solid line with square markers), while the maximum reduction was observed for *Ku* = 1 and *Vhalf* = −1 (see solid line with circle markers). The ranges of *Ku* (1e−5 to 1) and *Vhalf* (−1 to 200) were set based on the bounds of the parameters in the 28 “CiPA drugs.”

**Table 2:** Ranges of input parameters.

Parameters	Min	Max	Description
*bINa*, %	0	80	Percent block of fast sodium current
*bINaL*, %	0	80	Percent block of late sodium current
*bIto*, %	0	80	Percent block of transient outward current
*bIKs*, %	0	80	Percent block of slowly activating delayed rectifier potassium current
*bICaL*, %	0	80	Percent block of L-type calcium channel current
*bIK1*, %	0	80	Percent block of inward rectifier potassium current
sbIKr	0	4	Static component of the hERG channel current
*Vhalf*, mV	−200	−1	Degree of drug trapping for the hERG channel
*Ku*, ms^−1^	0	1	Unbinding reaction rate for the hERG channel

As for the other channel currents (i.e., fast sodium current *INa*, late sodium current *INaL*, L-type calcium channel current *ICaL*, slow-rectifying potassium channel current *IKs*, inward rectifier potassium current *IK*1, and transient outward current *Ito*), we used the general Hill equation of channel block,

(2) $$b_{\mathrm{current,drug}}=100\%\times \frac{C_{\mathrm{drug}}^{h}}{IC_{50,\mathrm{current}}^{h}+C_{\mathrm{drug}}^{h}},$$

where current = {*INa*, *INaL*, *ICaL*, *IKs*, *IK*1, *Ito*}, *IC*_50,_*_current_* is the drug concentration at which a current is reduced by half, *C_drug_* is the drug concentration, and *h* is the Hill coefficient. The drug-induced blocks of channel currents *b*_*current*,*drug*_ are used to scale the maximum conductance of the current *g_current_* in the *in silico* model calculated as

(3) $$g_{\mathrm{currennt,drug}}=\frac{100\%-{\text{b}}_{\mathrm{current,drug}}}{100\%}\times g_{\mathrm{current}}$$

We perform GSA explicitly with respect to *b*_*current,drug*_ rather than *IC*_50,*current*_, *C_drug_*, and *h*. In this study, we refer to the parameters of the block of *INa*, *INaL*, *ICaL*, *IKs*, *IK*1, and *Ito* as *bINa*, *bINaL*, *bICaL*, *bIKs*, *bIK*1, and *bIto*, respectively. Equation (2) is used in classification of real compounds.

### Sampling Virtual Drug Populations

A first population of virtual drugs (n = 10,000) was generated *via* Saltelli’s sampling scheme (Saltelli, 2002) over a nine-dimensional input parametric space describing drug binding and blocks of ionic currents, which we refer to in the manuscript as Virtual Drug Population I. The Saltelli’s scheme extends Sobol sequences resulting in samples that are almost uniformly distributed over the parameter space (see the **Supplemental Material** for the marginal and joint probability distributions of virtual drug parameters). The parameter ranges considered for generation of Virtual Drug Population I are listed in **Table 2**.

**Figure 2:**
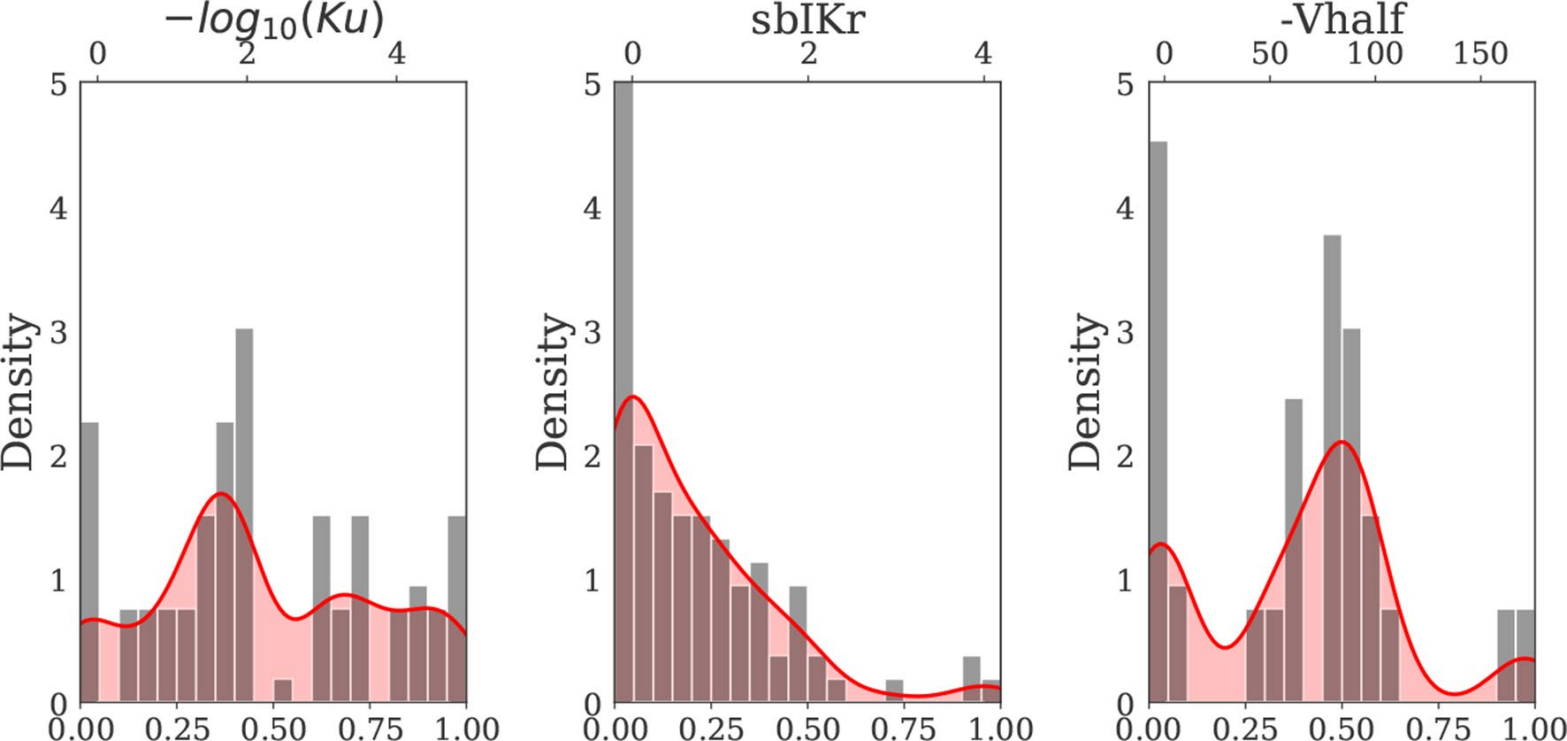
Kernel density estimate of the hERG channel parameters *Ku*, *sbIKr*, and *Vhalf* (solid red curve) constructed based on the distribution of the 28 “CiPA drug” parameters (gray bars) (Dutta et al., 2017; Li et al., 2017). MinMax normalization was performed for each input parameter prior to kernel density estimation. The x axis on the top of each plot indicates the actual (denormalized) parameter ranges for each of three hERG channel parameters.

Since parameter ranges were conservatively chosen to cover many possible combinations of current blocks, the actions induced by known drugs were located within small subregions of the larger parametric space. To gain further insights on the drugs belonging to these subregions of interest, we then generated a second virtual drug population (n = 10,000) based on the prior distribution of each of the nine parameters for the 28 “CiPA drugs” [calculated at 1–4× effective free therapeutic plasma concentration (EFTPC)], which we refer to as Virtual Drug Population II. Samples were generated *via* kernel density estimation under the assumption of independent ion channel blocks. Kernel density estimation allows to approximate the probability density function of any random variable given a finite data sample. For the procedure, we performed MinMax normalization for each of the parameters and used a Gaussian kernel with a standard deviation of 0.08. The samples (virtual drugs) with parameter values outside the range prescribed by the “CiPA drugs” were discarded. The marginal distribution of the hERG channel parameters of the “CiPA drugs” and the marginal distribution of the hERG channel parameters of the virtual drugs generated by kernel density estimation are shown in **Figure 2**. The marginal and joint probability distributions for each of the nine examined parameters are reported in the **Supplemental Material**.

### *In Silico* Simulations and Derived Features

The cell action potential and calcium transients were simulated for the two virtual populations of drugs generated for GSA and, separately, for the CiPA training (12 drugs) and validation (16 drugs) datasets (Dutta et al., 2017; Li et al., 2017). Simulations were run on the CiPAORd endo cell model. Model simulations were run for 1,000 beats to achieve a steady state. The simulations were initialized from control (no drug) steady-state values and were carried out at a pacing rate of 2,000 ms to simulate bradycardia unless explicitly specified. The CiPAORdv1.0 model code accessible at https://github.com/FDA/CiPA (Chang et al., 2017) was used with minor modifications introduced at the interface level to perform simulations in the study. Briefly, model equations were written in C and compiled for access by the R programming language (version 3.2.3). The system of ordinary differential equations (ODEs) was then solved using the *lsoda* solver from the *deSolve* R package (version 1.21) with both relative and absolute error tolerances set to 10^–6^. Model-derived metrics listed in **Table 3** were calculated from the action potential and the *Ca*^2+^ transients. The last five beats of a simulation were analyzed to extract derived features. Note that the metric *qNet* was calculated as the area under the curve traced by the net current (*Inet* = *ICaL*+*INaL*+*IKr*+*IKs*+*IK*1+*Ito*) from the beginning to the end of the last simulated beat as defined in Dutta et al. (2017).

**Table 3:** Derived features extracted from CiPAORd model.

Derived Feature	Description	Units
*qNet*	Net electronic charge carried by *IKr*, *INaL*, *ICaL*, *IKs*, *IK1*, *Ito* currents	nC/µF
*APD90*	Action potential duration at 90% repolarization	ms
*APD50*	Action potential duration at 50% repolarization	ms
*peakVm*	Peak voltage	mV
*diastolicCa*	Diastolic calcium level	nM
*peakCa*	Peak value of intracellular calcium	nM
*CaTD50*	Calcium transient duration at 50% return to baseline	ms
*CaTD90*	Calcium transient duration at 90% return to baseline	ms

### EAD Protocol

Drug-induced EAD risk (i.e., the sensitivity of a cell model against EAD generation) was evaluated for both virtual drug populations and for the “CiPA drugs” in the endo cell model. The EAD risk of a drug was evaluated *via* estimation of the amount of hERG channel perturbation (i.e., reduction in its maximum conductance) required to generate EADs in addition to the drug-induced parameter changes. This protocol of EAD risk estimation was previously proposed in Dutta et al. (2017). In the paper, we refer to the estimate of additional perturbation as *Th_EAD ,hERG_* metric. Simulations were run for varying degrees of *IKr* conductance reduction ranging from 0% to 100% with steps of 0.5%. The occurrence of EADs in a simulation was determined by analyzing the voltage trace of the last five beats. Beats with positive voltage differential (dV/dt) during the plateau phase of the AP were defined as carrying EADs and were detected by the code provided at https://github.com/FDA/CiPA (Chang et al., 2017).

### GSA

GSA was performed using a variance-based sensitivity method (Sobol’, 2001; Saltelli et al., 2008), and Monte Carlo filtering (MCF) (Hornberger and Spear, 1981; Saltelli et al., 2008).

#### Variance-Based GSA

Sobol sensitivity analysis method (Sobol’, 2001) is a model-independent GSA method that is based on variance decomposition. It relies on an all-at-a-time sampling strategy where output variations are induced by varying all the input factors simultaneously. Let a derived-metric *Y* from a computational model be represented by a function *f* of the input parameters,

(4) $$Y=f(\text{X})=f(X_{1},X_{2},\cdots ,X_{k}),$$

where **X**={*X*_1_,*X*_2_⋯*X_k_*} is the input parameter set. The function can then be decomposed into a sum of elementary functions of increasing dimensions,

(5) $$Y=f_{0}+\sum_{i}f_{i}(X_{i})+\sum_{i}\;\sum_{j>i}f_{ij}(X_{i},X_{j})+\cdots +f_{12\cdots k}(X_{1},\cdots ,X_{k}).$$

The input parameters are assumed to be random variables that are uncorrelated and mutually independent. The functional decomposition can be translated into a variance decomposition. This allows to quantify the variance contribution to the total output of individual parameters and the parameter interactions,

(6) $$V(Y)=\sum_{i}V_{i}+\sum_{i}\;\sum_{j>i}V_{ij}+\cdots +V_{123\cdots k},$$

where $V_{i}=V_{X_{i}}[E_{X_{∼i}}(Y|X_{i})]$ is the first-order effect for a given model input *X_i_*, $V_{\mathrm{ij}}=V_{X_{i},}{\;}_{X_{j}}[E_{X_{∼\mathrm{ij}}}(Y|X_{i},X_{j})]-V_{X_{i}}[E_{X_{∼i}}(Y|X_{i})]-V_{X_{j}}[E_{X_{∼j}}(Y|X_{j})]$, and so on are the higher-order effects. Here, $E_{X_{i}}$, $V_{X_{i}}$ are expectation and variance taken over *X_i_*; *X*_∼_ *_i_* denotes all factors but *X_i_*. The Sobol sensitivity indices are obtained as the ratio of partial variance to the total output variance,

(7) $$\begin{array}{cc} S1_{i}=\frac{V_{i}}{V(Y)}, & S2_{\mathrm{ij}}=\frac{V_{\mathrm{ij}}}{V(Y)}\ldots \end{array}$$

The number of sensitivity indices in (7) grow exponentially with *k*, and typically only sensitivity indices of up to order 2 (*S*1*_i_* and *S*2*_i_*) and the total-effect indices (*ST_i_*) are estimated (Iooss and Lemaître, 2014). The total-effect index

(8) $$ST_{i}=\frac{E_{X∼i}[V_{X_{i}}(Y|X_{∼i})]}{V(Y)}=1-\frac{V_{X∼i}[E_{X_{i}}(Y|X_{∼i})]}{V(Y)}$$

measures the impact of the main effect of *X_i_* and all its higher-order effects (Homma and Saltelli, 1996). The Python SALib package was employed to perform the variance-based sensitivity analysis (Herman and Usher, 2017). The calculations of *S*1*_i_*, *ST_i_*, and *S*2*_ij_* require *n*×(2*k*+2) model evaluations using Saltelli’s sampling scheme (Saltelli, 2002) where *n* is the sample size and *k* is the number of input parameters. In this study, we considered *n* = 500 unless otherwise specified, resulting in 10,000 Monte Carlo samples (virtual drugs) for *k* = 9.

Multivariate linear regression has been used in the past (Sobie, 2009) to identify sensitivity of outputs from cardiac cell models to changes in input parameters. In the **Supplemental Material**, we compare this standard linear regression technique ^1^ to the variance-based sensitivity analysis adopted in this paper.

#### MCF

MCF is generally used in factor-mapping tasks to identify key input parameters responsible for driving model outputs within or outside predefined target regions [refer to Saltelli et al. (2008) for a detailed description of the methodology]. Here, we present a brief overview of the MCF technique in the context of EAD sensitivity analysis of the CiPAORd endo cell model. After carrying out model simulations for the two virtual drug populations under the additional hERG perturbations required to induce EADs (see section EAD Protocol), samples were classified as either “Behavioral” (*EAD*−) or “Non-behavioral” (*EAD*+) based on the absence or presence of EADs in their simulated outputs, respectively. In other words, for each virtual drug population, the *n* samples were distributed between a “Behavioral” subset of *n*_1_ elements and a “Non-behavioral” subset of *n*_2_ = *n* − *n*_1_ elements. For each input parameter, *X_i_*, we then constructed empirical cumulative distribution functions (CDFs) of “Behavioral,” $F_{n_{1}}(X_{i}|\mathrm{EAD}-)$, and “Non-behavioral” samples, $F_{n_{2}}(X_{i}|\mathrm{EAD}+)$. The distance between these two empirical CDFs provides an estimate of the sensitivity of *EAD* generation to variations in *X_i_*. We used the Kolmogorov-Smirnov two-sample test statistic to quantify a D-statistic for the CDF distance and a p-value for the confidence of the estimate (Saltelli et al., 2008). The D-statistic is defined as

(9) $$d_{n_{1},}{\;}_{n_{2}}=\mathrm{maximum}||F_{n_{1}}(X_{1}|\mathrm{EAD}-)-F_{n_{2}}(X_{i}|\mathrm{EAD}+)||.$$

The larger the D-statistic (or equivalently the smaller the p-value), the more important the input parameter is in driving the behavior of the model towards EAD (Saltelli et al., 2008). The sensitivity of *EADs* to the different input parameters has been recently analyzed using multivariate logistic regression (Morotti and Grandi, 2016). Unlike logistic regression, which provides an accurate sensitivity measure when a hyperplane is able to separate the sub-regions of interest in the parameter space, the MCF method is valid even in more general cases, where the sub-regions of interest can be delineated only by highly non-linear or discontinuous surfaces (see **Supplemental Material** for comparison of the MCF and logistic regression methods on simple examples). In the absence of prior knowledge about the linearity of the surfaces separating the low, high, and intermediate drugs, we thought that the MCF method would be more appropriate. Moreover, MCF presents a better choice for estimating sensitivity indices for non-uniformly distributed data such as our Virtual Drug Population II. EAD sensitivity of the endo cell model was estimated at two different thresholds of additional hERG perturbations, which were inferred from the analysis of the 28 “CiPA drugs.” This helped us identifying the critical channels that allow separating the virtual drugs into high-, intermediate-, and low-risk groups. Further details of the analysis and threshold values used are provided in the Results section.

In addition, we also applied MCF to identify key input parameters responsible for separating virtual drugs into low-, intermediate-, and high-risk groups based on the *qNet* metric. Briefly, for each virtual drug population, the *n* samples were categorized into low- (*qNet* < *th*1), intermediate- (*th*1 ≤ *qNet* < *th*2), and high-risk (*qNet* ≥ *th*2) groups, based on the output *qNet* value. Empirical CDFs were then estimated for all input parameters and for all three categories of samples. Measures of distance between the low- and high-risk subset CDFs and between the high- and intermediate-risk subset CDFs were estimated using the Kolmogorov-Smirnov test as was also performed for the EAD sensitivity analysis.

#### Mean Decrease Accuracy

We also applied the Mean Decrease Accuracy (MDA) method for estimation of sensitivity. MDA or permutation feature importance is a commonly used machine-learning technique to rank the features. MDA is a model-agnostic method that can be applied to both classification and regression models. It was originally introduced to identify feature importance in random forest (Breiman, 2001). The importance of the features is evaluated individually by measuring the decrease in performance of the classifier/regression model after random permutation of the particular feature. In the context of sensitivity analysis of model-derived metrics, we first build a surrogate of the model-derived metric by fitting a linear or non-linear machine learning regression model (e.g., linear regression model, random forest regressor model, etc.) between the input parameters (**Table 2**) and the model-derived metric. Once trained, the model is fixed, and the performance (e.g., by *R*^2^ score in case of linear regression and random forest regressor models) is re-evaluated on modified input datasets obtained by randomly shuffling value entries of each of the parameters one at a time. Model performance is most sensitive to random permutations of important parameters. In this case, the method is used as an alternative to Sobol sensitivity. Similarly, for metrics with categorical values, we build a surface separating the different classes by fitting a machine-learning classifier model (e.g., logistic regression, random forest classifier model, etc.). The sensitivity of metrics with categorical values is estimated by calculating the decrease in classification accuracy on random shuffling of input parameters. In this case, the method is used as an alternative to MCF. We used Python’s scikit-learn package (Pedregosa et al., 2011) to train/test the different machine-learning models. In the **Supplemental Material**, we provide comparison of the sensitivity estimates obtained by the different methods in evaluation of simple hypothetical examples.

### Tertiary Risk Stratification of “CiPA Drugs”

*In silico* simulations of blocks with the 28 “CiPA drugs” were carried out using the *in vivo* manual patch clamp measurements collected on the pharmacological effects of these compounds reported in Li et al. (2017, 2018). The effective therapeutic concentrations, the *IC*_50_ values, the Hill coefficient values, the drug binding parameters, and the defined torsadogenic risk of the “CiPA drugs” are listed in the **Supplemental Material**. “CiPA drugs” were simulated at four different concentrations ranging from 1×to 4× EFTPC. “CiPA drugs” were also simulated using protocols described in the EAD Protocol section at progressively increasing hERG channel perturbations (0–100% block). The “CiPA drugs” were classified based on the amount of additional hERG channel perturbations required to induce EADs in the CiPAORd endo cell model as in Dutta et al. (2017). The classification of the “CiPA drugs” based on the *qNet*, *APD*90, and *peakCa* metrics was also performed for comparison. The threshold values necessary to optimally separate the drugs into different groups were estimated *via* logistic regression.

## Results

### GSA of Model-Derived Metrics: *APD90*, *qNet*, and *peakCa*

#### Variance-Based Analysis

**Figure 3:**
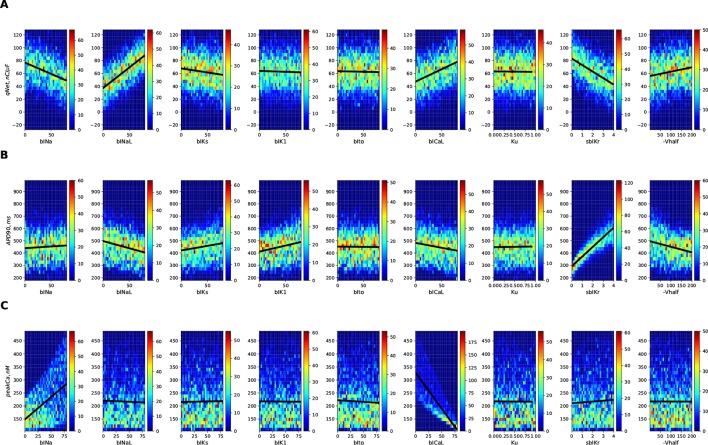
2D histogram plot of **(A)** *qNet*, **(B)** *APD90*, and **(C)** peak Ca metrics as a function of different input parameters (direct features) for the 10,000 drugs of Virtual Population I simulated in the endo cell model.

**Figure 3** shows the 2D histogram distribution of *qNet*, *APD*90, and *peakCa* values collected from the 10,000 simulations corresponding to the first (uniformly distributed) population of virtual drugs (Virtual Drug Population I). The estimated *qNet*, *APD*90, and *peakCa* values are plotted against individual input parameters to show their relative influence. In general, comparable blocks of a particular ion channel could result in significantly different output responses due to concomitant effects from other input parameters, as shown by the variability along the Y axis. Best-fit regression lines modeling relationship between the output metrics and the individual parameters are also added to the plots. We observed clear trends such as the increase in *APD*90 with the *sbIKr* parameter, the decrease in *peakCa* with the increase in the *bICaL* parameter, and increase in *qNet* with block of late sodium current.

**Figure 4:**
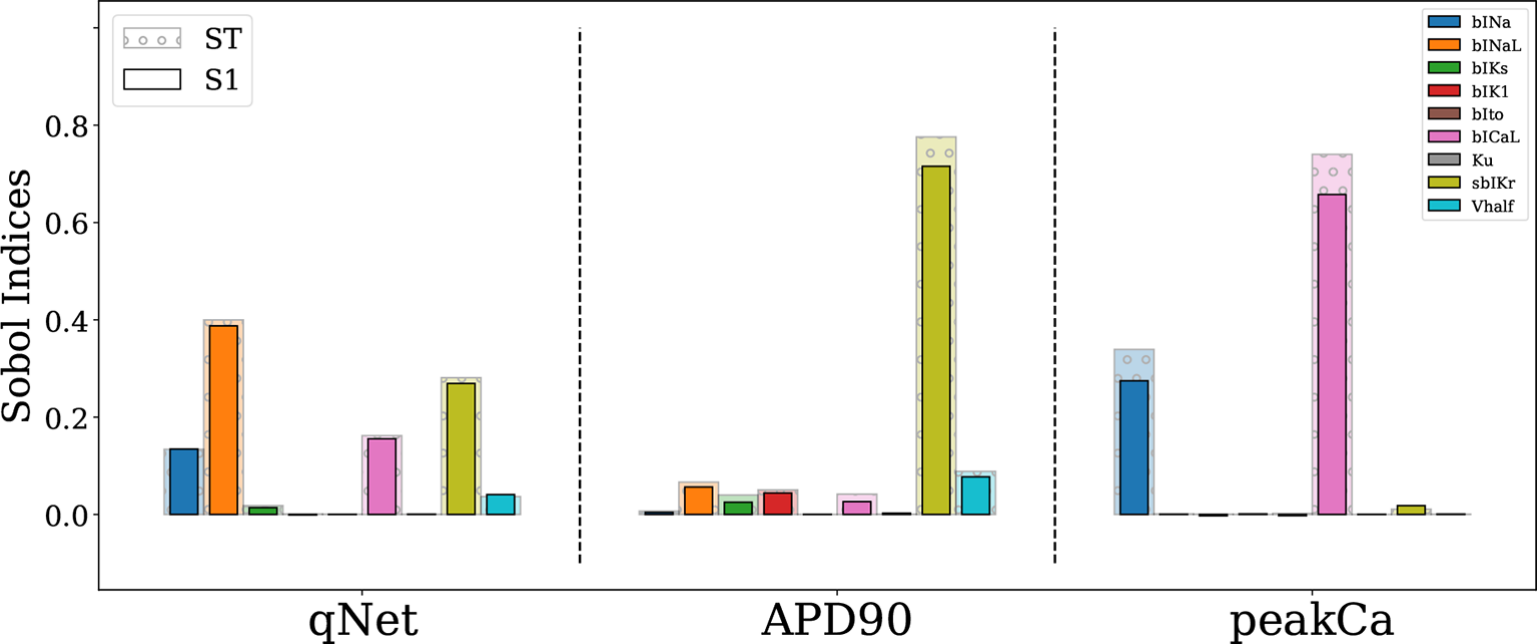
Sensitivity of *APD*90, *qNet*, and *peakCa* output responses to blocks of different cardiac ion channels and drug binding parameters in the CiPAORd endo cell model estimated *via* the Sobol method. The solid bars indicate the first-order sensitivity Sobol index, *S*1, and the transparent bars with circular patches show the estimated total sensitivity Sobol index, *ST*.

The Sobol sensitivity indices quantify the influence of individual parameters on the derived metrics. **Figure 4** shows values of the first-order Sobol sensitivity indices (*S*1, solid bars) and total sensitivity indices (*ST*, transparent bars with circular patches) for the same three output responses: *APD*90, *qNet*, and *peakCa* simulated in the CiPAORd endo cell model. The Sobol sensitivity indices indicate that *APD*90 is the most sensitive to *sbIKr* block, *qNet* to *sbINaL*, and *peakCa* to *bICaL*. The effect of *sbIKr* on *APD*90 as quantified by *S*1 indicates that *sbIKr* contributes more than 70% of the variation observed in *APD*90 across the observed input space. *qNet* was found to be most sensitive to *bINaL*, *sbIKr*, *bICaL*, and *bINa* with contributions to the output variation of 40%, 26%, 16%, and 13%, respectively. *bICaL* had the strongest impact on the variability of *peakCa* concentrations with an *S*1 index of around 0.6. Among the different drug effects evaluated *via in vitro* ion-channel screening, the changes in the block of transient outward current and dynamic hERG kinetic parameters showed relatively minor influences on the tested model-derived metrics. Small differences between *S*1 and *ST* for several derived metrics such as *APD*90 and *qNet* suggest minor influence of higher-order effects (**Figure 4**). The *S*1 and *ST* sensitivity indices obtained for the other model-derived features (**Table 3**) are reported in the **Supplemental Material**. The **Supplemental Material** also presents sensitivity analysis results obtained using multivariate linear regression methods.

The *R*^2^ value of a linear regression fit indicates the proportion of the variance in the dependent variable that is predictable from a linear combination of the independent variables. The estimated *R*^2^ values of multivariate linear regression fits for different model-derived metrics are listed in **Table 4**. The observed values indicate that >90% of the variance in *qNet*, *APD*90, and *peakCa* can be attributed to the linear combination of input parameters. Nonlinear terms explain less than 10% of the variance of these derived metrics. Moreover, metrics such as *qNet*, *APD*50, *APD*90, and *diastolicCa* exhibited *R* ^2^ values greater than 0.94. The *CaTD*90 and *peakCa* were the only features that had *R* ^2^ values of less than 0.91. This was in agreement with our Sobol analysis where the *ST* index showed relatively higher values compared to the *S*1 index for both these features, indicating the role of higher-order terms. Further analysis suggested that the role of second-degree interactions is minimal (results not shown), thus pointing towards discrepancies between *S*1 and *ST* attributable to even higher-order terms.

**Table 4:** Proportion of the variance in derived metrics explained by the first order terms (i.e. the input parameters) as estimated by *R* *^2^* value of multivariate linear regression.

	*qNet*	*APD90*	*peakC*a	*APD50*	*diastolicCa*	*CaTD50*	*CaTD90*	*peakVm*
*R^2^*	0.97	0.94	0.90	0.94	0.96	0.83	0.92	0.88

Importantly, we identified key differences among most influential parameters regulating different model-derived metrics. Specifically, *qNet* was the only metric sensitive to the block of late sodium current.

#### MDA Method

**Figure 5:**
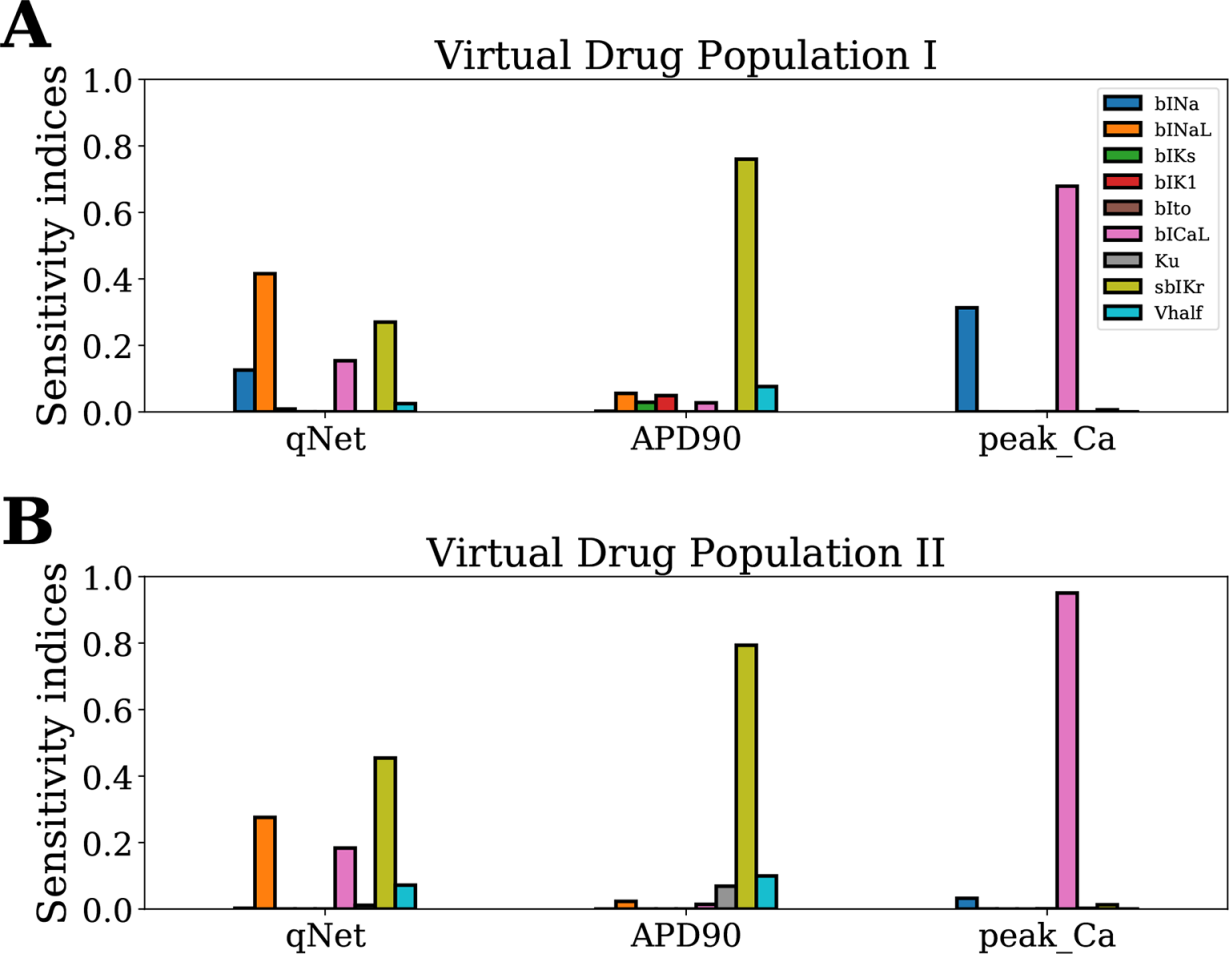
Sensitivity of *APD*90, *qNet*, and *peakCa* output responses to blocks of different cardiac ion channels and drug-binding parameters in the CiPAORd endo cell model estimated *via* the MDA method. **(A)** Virtual Drug Population I—10,000 virtual drugs sampled almost uniformly over the parametric space according to Saltelli’s scheme and **(B)** Virtual Drug Population II—10,000 virtual drugs sampled from a prior distribution based on the parameters for the 28 CiPA drugs.

Next, we analyzed the sensitivity of *APD*90, *qNet*, and *peakCa* for a second virtual population of 10,000 drugs (Virtual Drug Population II), which mimic more closely the “CiPA drugs.” However, estimation of Sobol indices in non-rectangular restricted domains is difficult and a topic of ongoing research (Kucherenko et al., 2017). Here, we used the MDA method to calculate the sensitivity indices of model-derived metrics for Virtual Drug Population II. A random forest regressor model (with hyperparameters *n_estimator_* = 100 and *max_depth_* = 12) was fit to each of the *APD*90, *qNet*, and *peakCa* metrics. The decrease in performance of the random forest regressor model was calculated on permutation of each input parameter. The trained models exhibited an excellent performance with a *R* ^2^ value >0.99 for each of the *APD*90, *qNet*, and *peakCa* metrics. **Figures 5A and B** show the sensitivity indices obtained for Virtual Drug Population I and Virtual Drug Population II, respectively. The sensitivity estimates obtained *via* the MDA method were similar to Sobol *ST* indices for the Virtual Drug Population I. We observed a modest difference in sensitivity profile of *qNet* between the two virtual populations. For example, the *qNet* metric was most influenced by *sbIKr* in the second virtual population. In contrast, *bINaL* was the most influential parameter for the Virtual Drug Population I. The sensitivity of *peakCa* to *bINa* was negligible for Virtual Drug Population II. Minor changes were observed in the sensitivity profile of *APD*90 across the two populations.

### Classification of CiPA Training/Validation Drugs Using Metric Based on EADs

**Figure 6:**
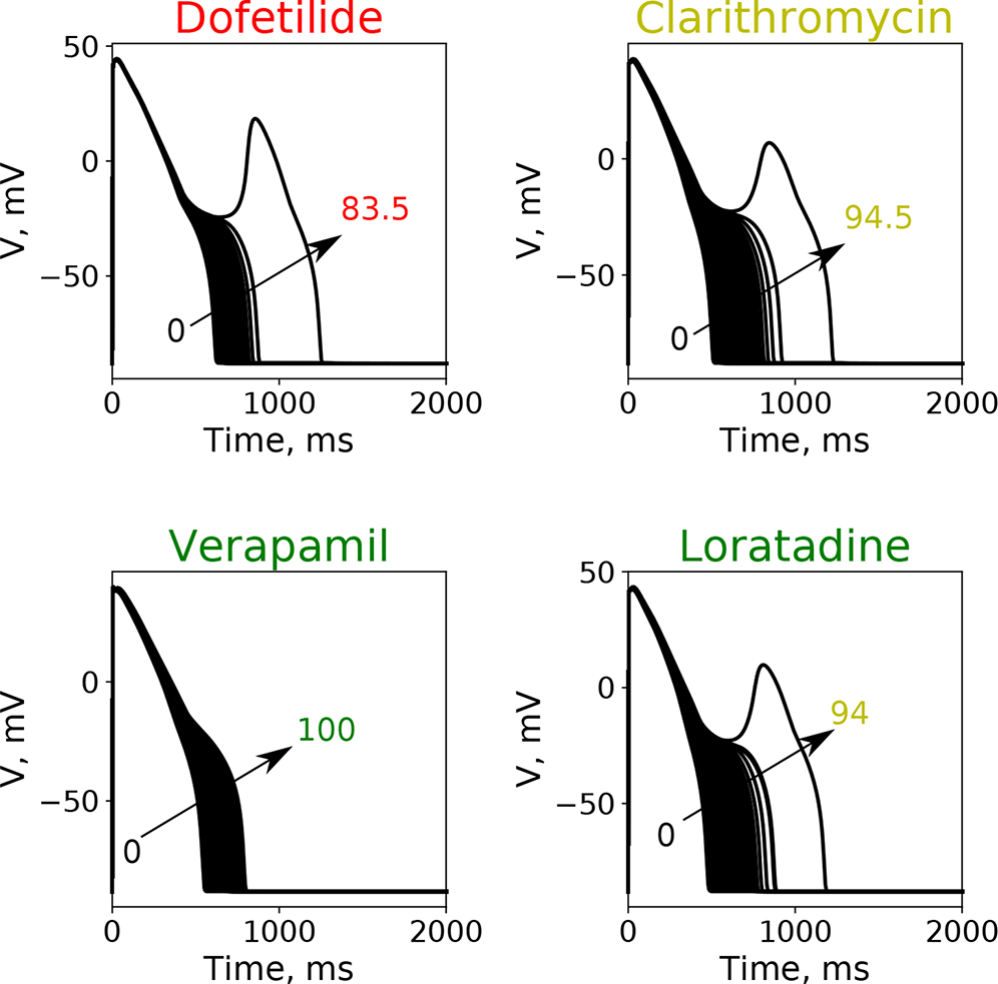
Typical action potential transients observed after the increase of additional block of hERG channel currents at a fixed drug concentration of 4× EFPTC in the endo cell model.

Here, we wanted to examine how the findings from the GSA on the virtual drug population would translate for the actual “CiPA drugs.” Moreover, we wanted to compare the performance of the simpler metrics *qNet*, *APD*90, and *peakCa* with respect to a metric based directly on EAD propensity. We evaluated the EAD development at drug concentrations between 1× and 4× EFTPC while increasing the additional block of hERG channels from 0% to 100%. **Figure 6** shows action potential traces obtained by simulating the EAD generation protocol for four representative “CiPA drugs” at 4× EFTPC. We observed that high-risk drug dofetilide is associated with EADs in the presence of relatively small additional perturbations of hERG current (84.5% block) compared to the low- and intermediate-risk drugs. The intermediate-risk drug clarithromycin and the low-risk drug loratadine resulted in generation of EADs in the presence of additional perturbations of hERG block around 94%. Verapamil did not generate EADs under any of the tested conditions.

**Figure 7:**
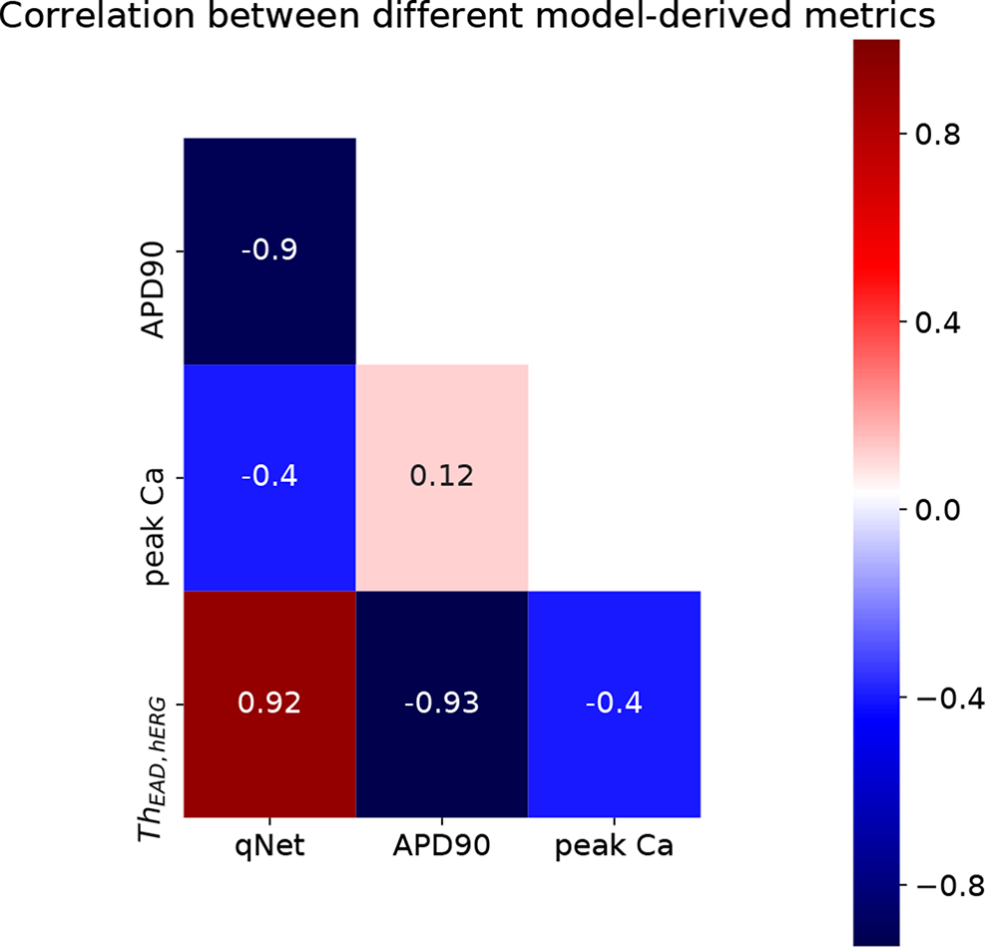
Heatmap of correlation between *qNet*, *APD*90, *peakCa*, and *Th_EAD,hERG_* metrics.

Using the above described protocol, we estimated the EAD metric (*Th_EAD,hERG_*) for all the 28 “CiPA drugs.” We also evaluated the *qNet*, *APD*90, and *peakCa* metrics for all the drugs at 1× to 4× EFTPC drug concentrations. The *Th _EAD,hERG_* metric was also estimated at 1× to 4× EFPTC drug concentrations. The mean metric values (average of the metric value estimated at 1–4× EFTPC) for each of the drugs is reported in **Table 5**. Next, we examined the correlations between these estimated metrics (*qNet*, *APD*90, *peakCa*, and *Th_EAD,hERG_* for the 28 “CiPA drugs” (**Figure 7**). In spite of the significant differences in the sensitivity profiles revealed by our GSA analysis, we observed that the metrics *qNet* and *APD*90 were highly correlated for the small dataset of 28 drugs. Moreover, we observed that the metrics *qNet* and *APD*90 also showed strong correlation with the *Th_EAD,hERG_* metric.

**Table 5:** Estimated values of the metric based on EADs, *qNet*, *APD90*, and *peakCa* for CiPA training (12 drugs) and validation (16 drugs) datasets.

Drug	*Th*_EAD_*_,hERG_*			*qNet* (*nC/µF*)	*APD*90 (*ms*)	*peakCa* (nM)	TdP risk
	endo cell			endo cell	endo cell	endo cell	
	C1		C2	C1	C1	C1	
Ibutilide	22.25		19.375	7.17	734	227	High
Quinidine*	15.62		28.12	20.80	775	206	High
Bepridil	87.625		84.25	44.59	424	229	High
Vandetanib	89.75		90.875	48.82	432	215	High
Azimilide	85.625		89.125	49.03	409	242	High
Dofetilide	87.5		88.87	51.83	376	242	High
Sotalol	89.375		90.0	56.05	363	248	High
Metoprolol	91.00		90.5	56.48	352	281	Low
Domperidone	99.625		99.625	59.91	382	163	Medium
Terfenadine	91.25		89.125	59.99	382	230	Medium
Cisapride	89.75		86.5	60.28	332	243	Medium
Droperidol	91.25		90.5	61.89	326	245	Medium
Ondansetron	91.00		91.125	62.10	340	238	Medium
Pimozide	92.75		89.625	62.14	334	215	Medium
Astemizole	92.00		92.125	62.97	318	243	Medium
Chlorpromazine	92.25		92.75	65.93	316	238	Medium
Clozapine	93.375		93.5	67.55	303	234	Medium
Tamoxifen	93.5		93.5	69.41	294	234	Low
Clarithromycin	94.00		93.875	69.56	302	220	Medium
Risperidone	93.75		93.5	70.23	290	232	Medium
Loratadine	93.75		93.75	70.44	289	233	Low
Disopyramide	95.000		95.0	72.64	288	213	High
Ranolazine	90.125		86.875	74.23	348	253	Low
Verapamil	99.25		99.125	74.85	320	157	Low
Nitrendipine	98.5		98.5	79.00	276	178	Low
Nifedipine	No EAD @ 100		98.5	87.77	261	142	Low
Diltiazem	No EAD @100		No EAD @ 100.0	92.05	257	130	Low
Mexiletine	97.625		94.75	92.26	304	200	Low
TdP risk classification summary							
	No. correctly classified		No. correctly classified		No. correctly classified	No. correctly classified	Total number of Drugs
Category	C1	C2	C1		C1	C1	
High	7 (4, 3)	6 (4, 2)	7 (4, 3)		6 (3, 3)	0 (0, 0)	8 (4, 4)
Intermediate	9 (3, 6)	7 (2, 5)	10 (4, 6)		6 (3, 3)	10 (4, 6)	11 (4, 7)
Low	5 (3, 2)	4 (2, 2)	7 (4, 3)		7 (4, 3)	5 (3, 2)	9 (4, 5)
Total	21 (10, 11)	17 (8, 9)	24 (12, 12)		18 (9, 9)	15 (7, 8)	28 (12, 16)

C1 Drug-induced modulation of nine parameters (sbIKr, Ku, Vhalf, bINa, bINaL, bICaL, bIKs, bIK1, and bIto) is considered.

C2 Only drug-induced changes in sbIKr and bICaL is considered (Vhalf = 100, Ku = 0.05).

* Quinidine resulted in EADs at drug concentrations greater than 2× EFTPC in the endo cell model. Hence, the qNet, Th_EAD,hERG_, APD90, and peakCa metric value reported for quinidine is average of the estimated metric at 1× and 2× EFTPC.

The red, yellow, and green colors in the drug column denote the high, intermediate, and low TdP risk drugs, respectively, as classified under the CiPA initiative. The metric values are colored red, yellow, and green depending on which risk group (high, intermediate, and low risk) the drug is classified into using the estimated metric.

Numbers in parentheses are number of drugs from training and validation set.

Next, we carried out classification of the 28 “CiPA drugs” into tertiary risk categories using the estimated *qNet*, *APD*90, *peakCa*, and *Th_EAD,hERG_* metrics. The two thresholds separating the drugs in the high-, intermediate-, and low-risk categories were obtained by applying logistic regression. The threshold values of 57 and 70 for the *qNet* metric were estimated for separation of the 28 “CiPA drugs” into three risk categories. These values are similar to those reported in Li et al. (2018) for the 16 CiPA training drugs. For the *Th _EAD,hERG_* metric, the two threshold values of 90 and 95 separated the “CiPA drugs” into high-, intermediate-, and low-risk groups. Threshold values of 307 and 367 were obtained for *APD*90. A threshold value of 204 was estimated for classification of low- and intermediate-risk drugs based on *peakCa*, which was not able to differentiate at all between intermediate- and high-risk drugs. We also built the *Th_EAD,hERG_* metric accounting only for the *sbIKr* and *bICaL* parameters (see **Table 5**).

Our EAD analysis shows that the drugs in the high-risk category consistently have a threshold value smaller than 90 for *Th_EAD,hERG_*, even when considering only drug effects on two parameters, *sbIKr* and *bICaL*. The addition of dynamic hERG channel current parameters as well as of other input parameters resulted in no significant changes in the observed thresholds for EAD generation. The high-risk drug disopyramide from the CiPA validation dataset did not induce EAD in the model under any of the tested conditions. Similarly, ranolazine and metoprolol drugs that are defined as low-risk under the CiPA initiative had a threshold value of less than 91 for *Th_EAD,hERG_*. The low-risk drugs diltiazem, verapamil, nifedipine, and nitrendipine resulted in EADs in the model only at threshold values greater than 95 under all of the tested conditions. Intermediate-risk drugs chlorpromazine, ondansetron, droperidol, astemizole, clozapine, clarithromycin, and risperidone resulted in EADs at relatively larger thresholds than high-risk drugs, >90 *Th_EAD,hERG_* but lower than the low-risk drugs, <95 *Th_EAD,hERG_*. The low-risk drug tamoxifen consistently resulted in EADs in the model at threshold values similar to intermediate-risk drugs. Pimozide, mexiletine, and terfenadine were among the only few drugs that switched risk category when the drug-induced changes of parameters other than *sbIKr* and *bICaL* were not considered.

**Figure 8:**
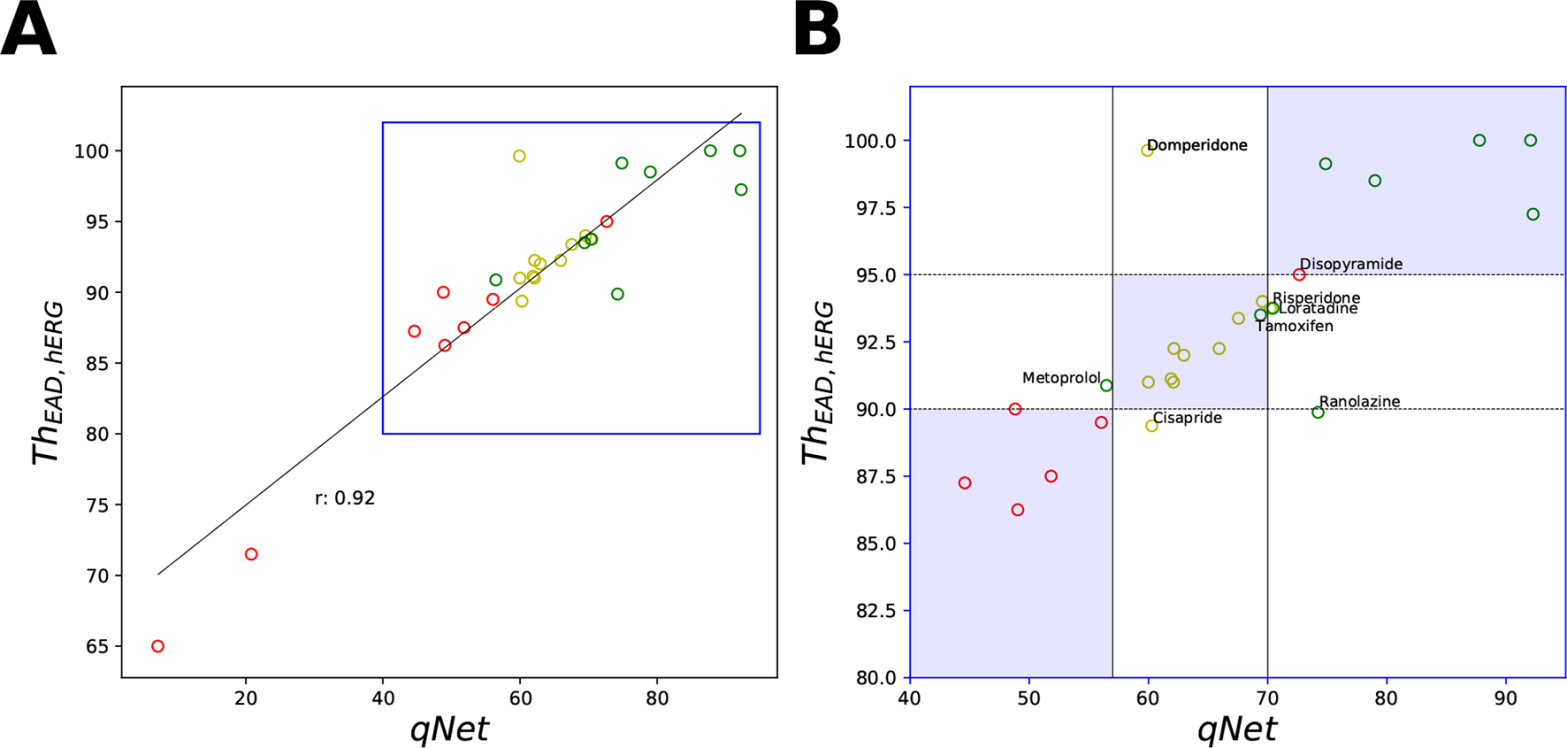
Scatter plot of *qNet* vs *Th_EAD,hERG_* metrics. **(A)** For all the 28 “CiPA drugs” a high correlation of 0.92 was observed. A region of interest is expanded in **(B)** to show details of separation among the drugs across the independently determined ranges for low, intermediate, and high risk based on *qNet* (solid black lines) and *Th_EAD,hERG_* (dotted black lines) metrics. Blue regions show where both the *qNet* and *Th_EAD,hERG_* metric agree. The high-, intermediate-, and low-risk drugs are colored in red, yellow, and green, respectively, based on their torsadogenic risk. See the **Supplemental Material** for an additional plot of *APD*90 vs *Th_EAD,hERG_* metrics.

Despite the high correlation among *APD*90, *qNet*, and *Th_EAD,hERG_*, we observed that *qNet* performed the best by classifying 24 of the 28 “CiPA drugs” correctly. *APD*90 correctly classified only 18 of the 28 drugs. The classifier based on EADs (*Th_EAD,hERG_*) alone instead correctly ranked only 21 drugs (**Table 5**). **Figure 8** shows a scatter plot of the best performing metric, *qNet*, against the metric directly based on simulated EADs, *Th_EAD,hERG_*, for the 28 “CiPA drugs.” The plot again shows the strong correlation between the two metrics and highlights out some of the misclassified drugs. Ranolazine, cisapride, domperidone, and loratadine were not correctly ranked based on the EAD metric but were instead correctly classified by *qNet*. On the contrary, only risperidone was correctly classified by *Th_EAD,hERG_* while also being narrowly misclassified based on *qNet*. Finally, the drugs metoprolol, tamoxifen, and disopyramide were not correctly classified by both metrics.

### Classification of Virtual Drugs *via* MCF

Here, we determine the most influential model parameters that allow separation of the virtual drug population into low-, high-, and intermediate-risk groups. The two populations of virtual drugs were partitioned into three different subsets based on the *qNet* and *Th_EAD,hERG_* thresholds obtained from analysis of the 28 “CiPA drugs.” For MCF, we constructed CDFs for each of the input parameters *X_i_* and for the low- (*X_i_*|*qNet* ≥ 70) intermediate- (*X_i_*|57 < *qNet* < 70), and high-risk (*X_i_*|*qNet* ≤ 70) subsets partitioned based on the estimated *qNet* metric. Similarly, the CDFs for each input parameter *X_i_* were calculated for the low- (*X_i_*|*Th_EAD,hERG_* ≥ 95), intermediate- (*X_i_*|90 *Th_EAD,hERG_* < 95), and high-risk (*X_i_*| ≤ 90) subsets based on the estimated *Th_EAD,hERG_* metric. The distance between the CDFs of the low- and intermediate-risk group and high- and intermediate-risk groups were estimated using Kolmogorov-Smirnov statistic. The estimated CDFs are shown in the **Supplemental Material**.

**Figure 9:**
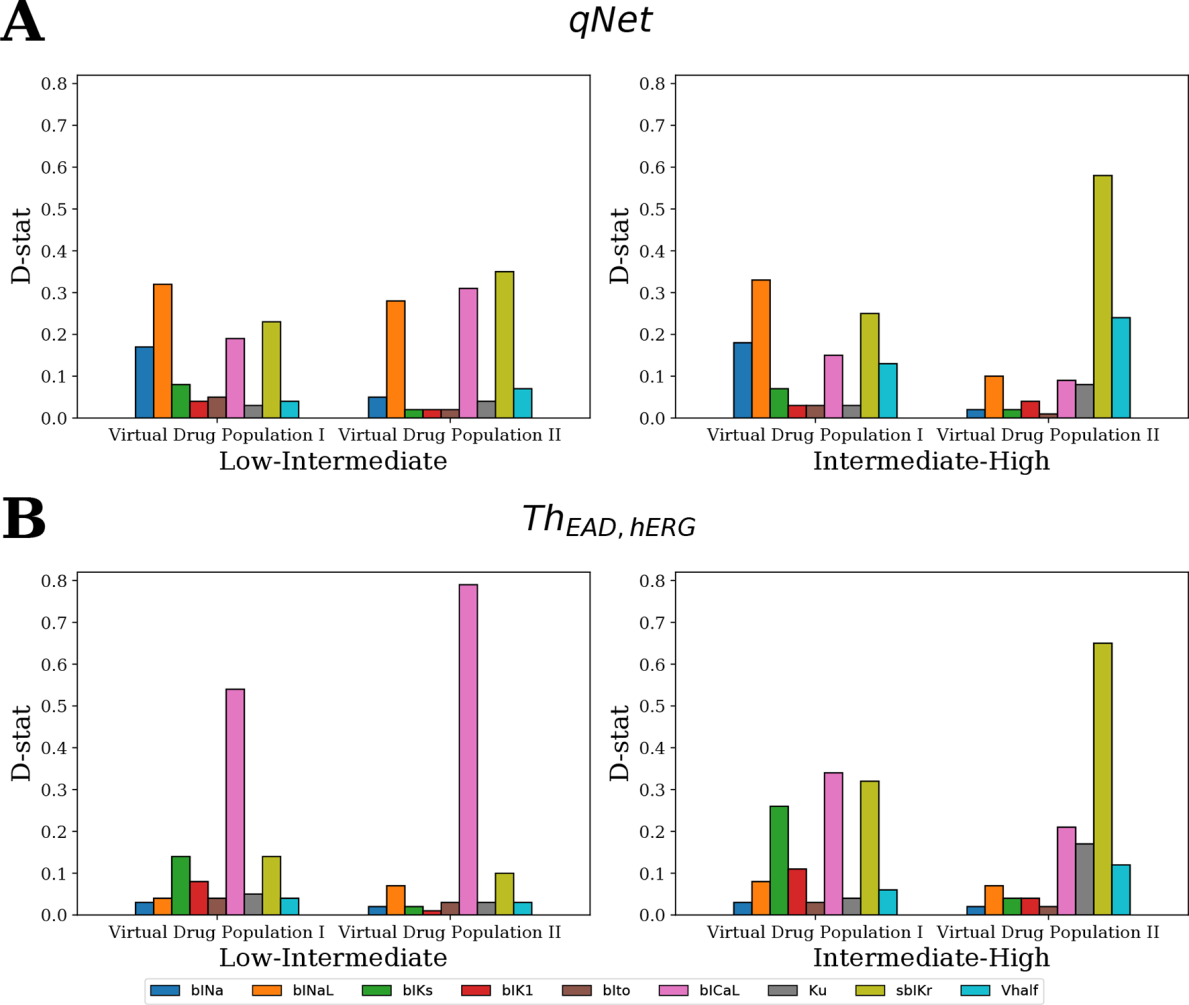
Ranking of the most influential model parameters for separating the virtual drugs into low-, high-, and intermediate-risk groups by MCF analysis. **(A)** Sensitivity measures for the separation of virtual drugs based on the *qNet* metric, and **(B)** based on *Th_EAD,hERG_* metric. Virtual Drug Population I—10,000 virtual drugs sampled almost uniformly over the parametric space according to Saltelli’s scheme. Virtual Drug Population II—10,000 virtual drugs sampled from a prior distribution based on the parameters for the 28 CiPA drugs.

**Figure 9** shows the D-statistic of the sensitivity estimates. For the first (uniformly distributed) virtual drug population (Virtual Drug Population I), our results show that the parameters *bINaL*, *sbIKr*, *bICaL*, and *bINa* had the highest influence in separating between the low- and intermediate-risk groups based on the *qNet* metric (**Figure 9A** left). Similarly, the parameters *bINaL*, *sbIKr*, *bINa*, *bICaL*, and *Vhalf* had the highest influence in separating the high- and intermediate-risk drugs (**Figure 9A** right). Both these results were in agreement with our Sobol sensitivity analysis (see **Figure 4**). On the contrary, *bICaL*, *sbIKr*, and *bIKs* (**Figure 9B**) were the most influential parameters in categorizing the drugs into low-, intermediate-, and high-risk groups based on the *Th_EAD,hERG_*.

We also analyzed a second virtual population (Virtual Drug Population II) of 10,000 drugs sampled from a distribution based on the 28 “CiPA drugs” (see Sampling Virtual Drug Populations section for further details). The parameters *bICaL*, *sbIKr*, and *bINaL* had the highest influence in separating the low- and intermediate-risk drugs based on the *qNet* metric (**Figure 9A** left). The parameter *bICaL* played the biggest role in separating the low- and intermediate-risk drugs based on the *Th_EAD,hERG_* metric (**Figure 9B** left). The most influential parameters for separation of high- and intermediate-risk drugs based on the *qNet* metric were *sbIKr* and *Vhalf* (**Figure 9A** right). The parameters *Ku* and *bICaL* also showed some influence in addition to the *sbIKr* and *Vhalf* parameters for separating between the high- and intermediate-risk drugs based on *Th_EAD,hERG_* (**Figure 9B** right).

In summary, the MCF analysis highlighted significant differences in the relative importance of input parameters depending on whether drugs were categorized according to *qNet* or to *Th_EAD,hERG_*. Interestingly, late sodium current block played an important role in risk discrimination based on the *qNet* metric, while its influence was only minor when the *Th_EAD,hERG_* metric was considered (**Figure 9**). This provides a possible explanation for the different risk categorization of ranolazine, a hERG and late sodium blocker, by the two metrics (**Figure 8**). Moreover, differences between results for the two virtual populations emphasize the importance of input parameter distribution in determining the sensitivity profiles (**Figure 9**).

## Discussion

Uncertainties in *in vitro* measurements of drug-induced effects on ionic currents present an important concern in evaluating the torsadogenic risk of compounds by interrogating *in silico* biophysical models. Discrepancies in estimates for model parameters based on available *in vitro* assay data have been recently highlighted in uncertainty quantification studies (Johnstone et al., 2016; Chang et al., 2017). High uncertainty in model parameters leads to low confidence in model predicted risk, and thus, not surprisingly, risk stratification of the CiPA training drugs proved to be unreliable especially at high drug concentrations (Chang et al., 2017), where model parameter estimates are inherently less accurate. However, it is important to emphasize that the relative contributions of drug-induced modulation of ion channels on output features differ significantly. Uncertainties in model input parameters that are highly influential (e.g., as revealed by sensitivity analysis) result, therefore, in lower confidence in the predicted risk, while errors in estimating less influential model parameters are better tolerated by risk measures (Mirams et al., 2016; Loucks et al., 2017). In this paper, we present a study that applies GSA within the context of *in silico* prediction of pharmacological toxicity. The target of GSA was the latest version of the *in silico* model of an isolated cardiac cell (Dutta et al., 2017), CiPAORd, which was developed under the CiPA initiative and incorporates dynamic hERG-drug interactions (Li et al., 2017). Our analysis explored the effects of a large population of virtual drugs on the seven major cardiac ion-channel currents thought to be important in regulation of TdP. GSA provided a systematic understanding of the model input-output relationships and allowed for the identification of the most influential parameters that regulate model-derived features used for proarrhythmic risk classification. The knowledge gained from GSA could help further improve the model structure and increase reliability of model-predicted risk.

### GSA of Output Metrics and Risk Classification

Different methods and tools, each with their own advantages and disadvantages, allow for the analysis of the sensitivity of complex systems to the input parameters [e.g., refer to (Saltelli et al., 2008; Iooss and Lemaître, 2014; Pianosi et al., 2016) for thorough reviews on the subject]. Simple sensitivity analyses performed by varying one parameter at a time have been carried out to asses the impact of changes in ionic currents on cardiac cellular electrophysiology (Romero et al., 2009; Chang et al., 2014). This type of sensitivity analysis, although computationally inexpensive, only quantifies the impact on model outputs of changes in a single input parameter relative to the point estimates chosen for the rest of the parameters that are held constant. On the contrary, GSA quantifies the effects of global variations over the entire input parameter space. Multivariate linear regression models that rely on all-at-a-time sampling approaches have been used in the past on the cardiac cellular models (Sobie, 2009) to identify how changes in model parameters affect different outputs of the model, to address different physiological questions, to improve model structure, and to suggest novel experiments (Sarkar and Sobie, 2010; Britton et al., 2013; Lee et al., 2013; Sadrieh et al., 2013; Cummins et al., 2014; Devenyi and Sobie, 2016; Devenyi et al., 2017). Recently, application of multivariate logistic regression has been reported to relate perturbations in model parameters to the presence/absence of EADs (Morotti and Grandi, 2016). The multivariate linear regression is suitable and accurate for models with almost linear input-output relationship. Similarly, the logistic regression applied to determine EAD sensitivity is accurate if a surface separating EAD and non-EAD regions is close to a hyperplane.

The prior distribution of model inputs is a critical factor that determines sensitivity of a model-derived metric to the inputs. Therefore, we tested two populations of virtual drugs, one sampled from a uniform distribution of blocks (Virtual Drug Population I) and another sampled from a non-uniform distribution representative of the blocks of the “CiPA drugs” (Virtual Drug Population II). Given the lack of prior knowledge about the behavior of certain model-derived metrics (e.g., *qNet* and EAD-based indices), we opted for using general forms of GSA that are suitable for non-linear input-output relationships (Saltelli et al., 2008). In particular, we used the Sobol variance-based sensitivity method (Sobol’, 2001; Saltelli et al., 2008) to rank cardiac ion-channel currents. However, we found that the *S*1 and *ST* indices are similar for most metrics, which indicates that these derived features can be almost fully recovered as linear combinations of channel blocks (see **Figure 4** and **Table 4**). Not surprisingly then, our sensitivity indices were similar to analogue coefficients computed *via* multivariate linear regression (see the **Supplemental Material** for comparison of the indices obtained with both methods).

The computation of Sobol *ST* indices is non-trivial when input parameters are not uniformly distributed (as for Virtual Drug Population II). Therefore, we employed an alternative GSA method, MDA, that gives a clear interpretation of feature ranking even for non-uniform distributions. In the **Supplemental Material**, we also show how, for a simple 2D case, MDA provides similar sensitivity estimates to the Sobol *ST* indices. To apply MDA, we first approximated the derived metrics by random forest metamodels. Then, we evaluated the accuracy of the metarepresentation upon random permutations of the values of a given feature. Losses in accuracy measured for each of the permutations provided us with global sensitivity estimates (**Figure 5**). Limitations of this method are (1) sensitivity estimates obtained *via* MDA rely on accuracy of the surrogate metamodel and (2) the performance of MDA methods suffers in case of strong correlation between inputs. For further comparison of different GSA methods on simple hypothetical examples, refer to the **Supplemental Material**.

**Figure 10:**
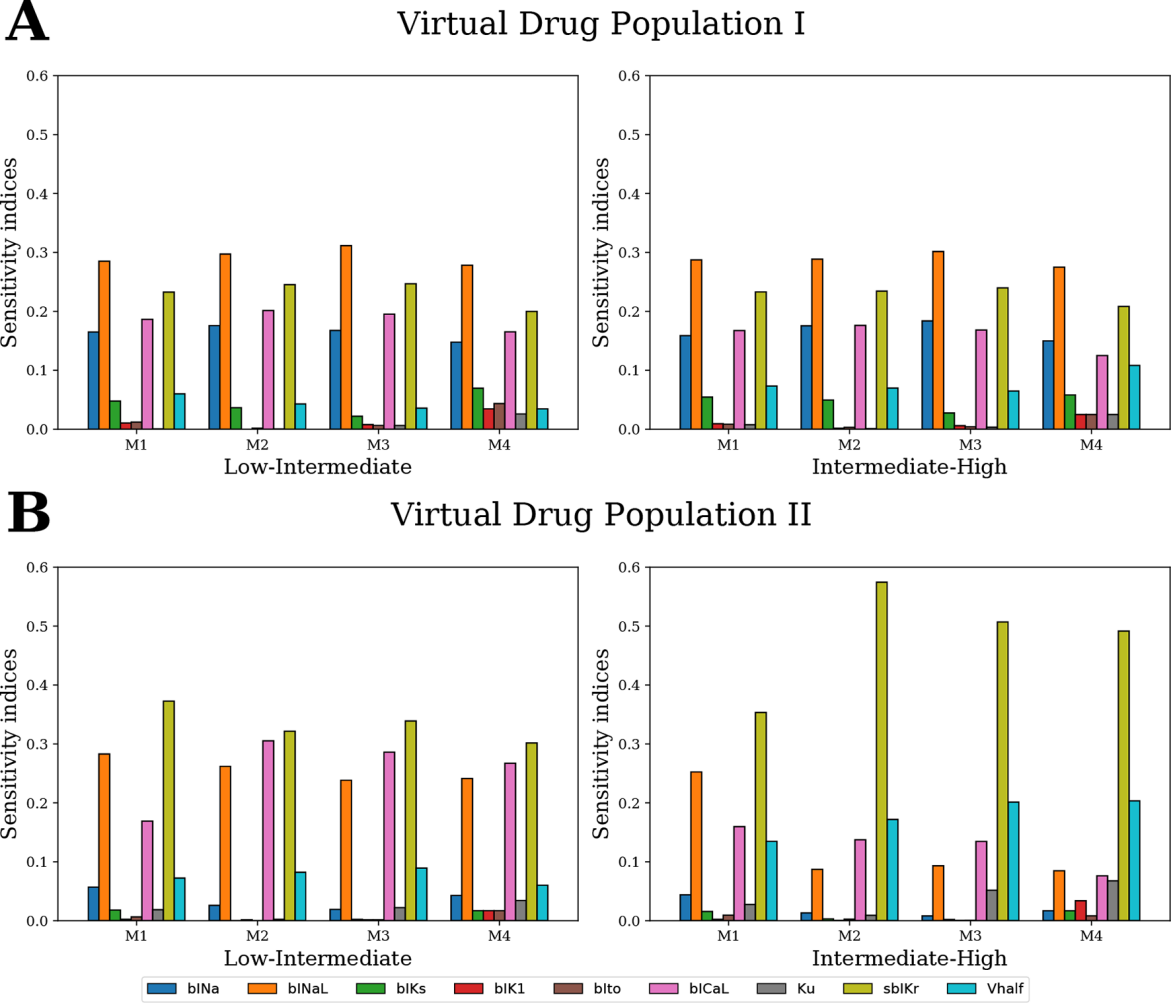
Ranking the most influential model parameters for separating the virtual drugs into low-, high-, and intermediate-risk groups by qNet via four different methods: M1–logistic regression method, M2–MDA of logistic regression, M3–MDA of random forest classifier, and M4–MCF. Sensitivity measures were estimated for **(A)** Virtual Drug Population I–10,000 virtual drugs sampled almost uniformly over the parametric space according to Saltelli’s scheme and **(B)** Virtual Drug Population II–10,000 virtual drugs sampled from a prior distribution based on the parameters for the 28 CiPA drugs.

For GSA of categorical outputs, we performed MCF (Hornberger and Spear, 1981; Saltelli et al., 2008). In particular, MCF was used to determine the cardiac ion channels that are most critical in drug classification. D-statistics from MCF were compared to sensitivity measures from logistic regression and MDA. For Virtual Drug Population I, which uniformly covers the entire parameter space, the results from all techniques were almost identical (**Figure 10A**). However, sensitivity estimates obtained for Virtual Drug Population II diverged (**Figure 10B**). In particular, for the non-uniformly distributed data, the sensitivity measures obtained by MCF and MDA methods were similar but differed significantly from the sensitivity estimates obtained from logistic regression. Differences in results are likely attributable to how these methods respond to biases introduced by nonuniform distributions, such as our second virtual population of drugs.

### Critical Inputs Regulating *qNet*, *APD90*, and *peakCa*

Our Sobol sensitivity analysis of the first virtual population of drugs identified critical input parameters for the variability of the different model-derived features used for TdP risk assessment (see **Figures 4** and **5** and data in the **Supplemental Material**). More specifically, we observed that the recently proposed *qNet* metric is most sensitive to modulations in sodium currents and to the *sbIKr* parameter. *sbIKr* was the most influential parameter regulating *APD*90 (**Figure 4**). In the past, *APD*90 has also been shown, by varying one parameter at a time in the original ORd model (O’Hara et al., 2011), to be most sensitive to a block of hERG current. Furthermore, the QT interval measured in 3D human heart simulations (Costabal et al., 2019) with original ORd model (O’Hara et al., 2011) at the cellular level exhibits a similar sensitivity profile to *APD*90. This is in agreement with previous observations of high correlation between *APD*90 and QT interval in the cardiac model simulations (Beattie et al., 2013). In our study, features derived from the calcium transient such as *peakCa* were found, as expected, to be most sensitive to the *bICaL* parameter. Interestingly, the recently introduced dynamic-hERG block parameters *Vhalf* and *Ku* (Li et al., 2017), which are measured using challenging experimental protocols (Milnes et al., 2010; Veroli et al., 2014), exhibit relatively small contribution to the variance of the *qNet*, *APD*90, and *peakCa* (**Figure 4** and data in the **Supplemental Material**). Moreover, several cardiac ion channels/parameters that are thought to be important for improved drug-induced TdP risk assessments and measured experimentally *via in vitro* ion-channel screening (Crumb et al., 2016) showed minor influence in regulation of the model-derived features. For example, the block of *Ito* and *IK*1 showed relatively minor influence on majority of the tested metrics (**Figures 4** and **5** and the **Supplemental Material**)

In spite of the above described differences in sensitivity profiles, several combinations of derived metrics have been reported to perform equally well in classifying the proarrhythmic risk of different drug datasets. For example, *APD*90 (Mirams et al., 2011), a metric based on *APD*50 and *diastolicCa* (Lancaster and Sobie, 2016) and a metric based on EADs (Christophe, 2013) have all been shown to provide good risk discrimination of drugs on the same dataset (Mirams et al., 2011). In addition, we have also shown previously that different derived features extracted from the original ORd model (O’Hara et al., 2011) show similar performance in TdP risk discrimination when tested on the combination of several datasets (Parikh et al., 2017). Overall, the similarity in performance might be due to measurements of drug effects being mainly limited to only three channel currents (i.e., fast sodium current, L-type calcium channel current, and hERG current), to the small size of the datasets, and to the differences in the myocyte model structure used to obtain the derived features. Indeed, *APD*50, *APD*90, *peakCa*, and *CaD*90 have been shown to provide the best classification when varying the computational model of interest (Mirams et al., 2011).

As a further analysis of the metric behaviors, we computed the intercorrelations between *qNet*, *APD*90, and *peakCa* for the 28 “CiPA drugs” (**Figures 7** and **8**). These compounds have been extensively characterized, and their actions on seven ion channels has been experimentally measured. To link the derived metrics to the physiological mechanism underlying arrhythmia, we also computed for each drug the additional hERG perturbation required to induce EADs (*Th_EAD,hERG_*). Our results indicate a strong correspondence between *qNet* and *APD*90, with a Pearson coefficient of −0.9. Both metrics, *qNet* and *APD*90, also correlated well with *Th_EAD,hERG_* (Pearson coefficient >0.9 and <−0.9, respectively). Not surprisingly, the correlations with *peakCa* and between *peakCa* and *Th_EAD,hERG_* were significantly lower (i.e., less than 0.4 in absolute value). In spite of strong correlation, the metrics showed different performance in drug classification. In particular, as in recent studies (Dutta et al., 2017; Li et al., 2018), *qNet* metric provided the most accurate proarrhythmic risk prediction (i.e, 24/28 correctly classified drugs) for the compounds in the CiPA dataset. We observed that *Th_EAD,hERG_* (21/28 correctly classified drugs), *APD*90 (18/28 correctly classified drugs), and *peakCa* (15/28 correctly classified drugs) performed worse than *qNet* (**Table 5**). While the worse classification performance of *peakCa* might have been expected given that it presents negligible sensitivity to *sbIkr*, the differences in performance among the other three metrics were not directly explainable. Therefore, we extended the classification analysis to a second virtual population of drugs chosen to be representative of the CiPA dataset as discussed in the sections below.

### Classification of Virtual Drugs Based on EAD Metric

MCF analysis revealed that the *bICaL* and the *sbIKr* parameters are the most influential for accurate classification of both populations of virtual drugs using the EAD-related metric (*Th_EAD,hERG_*) (**Figure 9**). The critical role of hERG channels in generation of EADs and eventually TdP (Redfern et al., 2003) is well established, and *IKr* is the primary current responsible for generation of EADs in the original ORd model (Christophe, 2013). The role of L-type calcium channel currents in regulation of EADs has been highlighted across different studies (January and Riddle, 1989; Zeng and Rudy, 1995; Weiss et al., 2010). The third most important parameter for EAD generation in the Virtual Drug Population I was *bIKs*. *IKs* has been previously shown to play an important role in EAD regulation (Devenyi et al., 2017). *bIKs* effects were instead almost negligible when considering the Virtual Drug Population II, which does not include many samples with blocked *IKs*. Note that *APD*90 and *qNet* were minimally sensitive to *IKs* for both virtual drug populations, suggesting that these metrics might be less suitable than *Th_EAD,hERG_* to categorize drugs with *IKs* block. Furthermore, drug-induced block of other cellular components such as sodium-calcium exchanger (*INaCa*) and sodium-potassium ATPase pump (*INaK*) currents could play an important role in accurate risk stratification of drugs using EAD-related metric. As previously found, these currents are important regulators of drug-induced repolarization abnormalities (Lancaster and Sobie, 2016; Passini et al., 2017). The analysis carried out in this study could be potentially updated to identify sensitivity of different metrics to the block of additional cellular components when experimental measurements for these additional parameters become available.

The dynamic hERG parameters (i.e., *Vhalf* and *Ku*) showed relatively minor contributions to classification of the Virtual Drug Population I. However, an increased role of the parameters was evident when tested on the Virtual Drug Population II, which mimics more closely the “CiPA drugs.” In addition, for both drug populations, the dynamic hERG parameters mainly improved the classification of high- and intermediate-risk drugs. These results support the finding of a previous study where the dynamic hERG parameters were introduced to achieve better risk discrimination between high- and intermediate-risk drugs Li et al. (2017). The results also highlight how MCF allows to individuate parameters that play an important role on restricted populations, while Sobol sensitivity tends to highlight those parameters that preserve importance throughout the entire sample population. For example, *Vhalf* affects only slightly the variability of *qNet* over the first virtual drug population as shown by Sobol sensitivity analysis (see **Figure 4**, where *Vhalf* is the fifth most important parameter), while the same input ranks as the second most important one when tested *via* MCF on classification of high- and intermediate-risk drugs from the second virtual population (**Figure 9**). It should also be noted that in cases where the majority of the primary regulating parameters are similar between drugs, accounting for changes in the modestly influential parameters can allow for improved predictions. On classifying CiPA drugs based on EADs, we observed that prediction improves by correctly classifying four more drugs when accounting for drug-induced effects of other parameters in addition to the *sbIKr* and *bICaL* parameters (**Table 5**). However, our results also point towards the important consideration that errors in measuring the most influential parameters regulating a particular metric have a bigger impact on the predicted classification compared to neglecting some of the less influential parameters. GSA allows us to determine and rank most of the critical model components.

### Mechanistic Insight From Model-Derived Metrics

Simple statistical classifiers based on direct feature from our group and others have been shown previously to provide equivalent performance as biophysically detailed models for TdP (Kramer et al., 2013; Mistry et al., 2015; Parikh et al., 2017; Mistry, 2018). Our sensitivity analysis results also highlight strong linearity between the inputs and different model-derived metrics (such as *qNet*, *APD*90, etc.) that are proposed for TdP risk stratification (**Table 4**). The metric linearity suggests that the model-derived metrics can be well captured as a linear combination of the set of direct features and provides a plausible explanation for equivalent performance of the simple statistical methods. Almost linear input-output relationship in different cardiac models has also been observed in several previous studies (Sobie, 2009; Sarkar and Sobie, 2010). However, one of the most appealing features for the biophysical models is that of interpretability, i.e., the model-derived features attempt to capture the aspects of the underlying physiological phenomena such as action potential duration (APD) prolongation or increase in calcium levels to provide a mechanism-based classifier. Being biophysically motivated, classifiers built on model-derived features are thought to allow generalizable assessments also in cases where the training datasets are small and hence the effects on targets of interest might need to be extrapolated. A promising metric *qNet*, proposed by the modeling team at FDA (Dutta et al., 2017), has recently been shown to provide excellent classification of drugs in the CiPA training and validation data, a result thought to be linked to EAD generation (Dutta et al., 2017; Li et al., 2018). However, our GSA results demonstrate that *qNet* and *Th_EAD,hERG_* have different sensitivity profiles (**Figure 9**) despite being highly correlated (**Figure 7**). While both metrics were sensitive to *sbIKr* and *bICaL*, only *qNet* was influenced by *bINaL*, a result maintained for both virtual populations of drugs. Moreover, we observed that the categorization of “CiPA drugs” based on analysis of EADs was not as predictive as *qNet* (**Table 5**). We found that drugs like ranolazine, cisapride, and domperidone, which were not correctly ranked by the EAD metric, were instead correctly classified by *qNet* (**Table 5** and **Figure 7**). Our analysis supports that *qNet* is able to classify ranolazine by accounting for the reduced TDR (Shimizu and Antzelevitch, 1998), which is affected by the block of the late sodium current. On the other hand, possible explanations for the poor performance of the EAD metric compared to *qNet* might include inaccurate reproduction of EADs in the current model, the type of EAD perturbations considered, the small size of the tested datasets, biases in the target, or the need to test EADs on coupled cells/tissue models.

### Summary

The proarrhythmic risk assessment based on simulated drug responses in isolated cell model (Mirams et al., 2011; Christophe, 2013; Trenor et al., 2013; Christophe, 2015; Lancaster and Sobie, 2016; Li et al., 2017; Dutta et al., 2017; Parikh et al., 2017; Passini et al., 2017; Li et al., 2018), tissue models (Kubo et al., 2017), or organ-level computational models (Okada et al., 2015; Costabal et al., 2018; Costabal et al., 2019) provide important physiological and mechanistic insights. Moreover, *in silico* models serve as an excellent tool for evaluation of drug safety in diseased conditions (Trenor et al., 2013; Kubo et al., 2017). However, the uncertainties in pharmacological data used for model-driven predictions and in the intrinsic structures of biophysical models used for cardiotoxic risk predictions present fundamental challenges. In this study, we showed potential application of sensitivity analysis for improved model-based proarrhythmic risk predictions. The critical model inputs regulating the model-derived metrics such as *APD*90 and *qNet* proposed for evaluation of proarrhythmic risk were identified. The analysis highlighted the need for better mechanistic understanding of promising metrics such as *qNet* and provided possible explanation for equivalent performance of the simple statistical-based classifiers and complex model-driven risk predictions. In conclusion, the sensitivity analysis method together with uncertainty quantification approaches can form an important component of the model-based cardiotoxic risk prediction pipeline. An improved pipeline would ultimately allow for refinement of existing biophysical models to achieve increased confidence in the model-driven proarrhythmic risk predictions.

## Data Availability

The datasets analyzed for this study can be found in the **Supplemental Material**.

## Author Contributions

JP designed the study, performed simulations, analyzed results, and wrote the manuscript. PA designed the study, analyzed the results, and wrote the manuscript. JK wrote the manuscript and supervised the project. VG designed the study, analyzed the results, wrote the manuscript, and supervised the project. All authors agree to be accountable for the content of the work.

## Conflict of Interest Statement

All authors are employees of IBM Research.

## Footnotes

1 Here and further in the paper, we discuss linear regressions with input features typically used in the sensitivity analysis of cell models, i.e., regressions with only linear combinations of features constructed from the input parameters.

## References

[B1] Beattie K. A. Luscombe C. Williams G. Munoz-Muriedas J. Gavaghan D. J. Cui Y. (2013). Evaluation of an *in silico* cardiac safety assay: using ion channel screening data to predict QT interval changes in the rabbit ventricular wedge. J. Pharmacol. Toxicol. Methods 68, 88–96. 10.1016/j.vascn.2013.04.004 23624022 PMC4142193

[B2] Breiman L. (2001). Random forests. Mach. Learn. 45, 5–32. 10.1023/A:1010933404324

[B3] Britton O. J. Bueno-Orovio A. Ammel K. V. Lu H. R. Towart R. Gallacher D. J. (2013). Experimentally calibrated population of models predicts and explains intersubject variability in cardiac cellular electrophysiology. Proc. Natl. Acad. Sci. 110, E2098–E2105. 10.1073/pnas.1304382110 23690584 PMC3677477

[B4] Chang K. C. Bayer J. D. Trayanova N. A. (2014). Disrupted calcium release as a mechanism for atrial alternans associated with human atrial fibrillation. PLoS Comput. Biol. 10, e1004011. 10.1371/journal.pcbi.1004011 25501557 PMC4263367

[B5] Chang K. C. Dutta S. Mirams G. R. Beattie K. A. Sheng J. Tran P. N. (2017). Uncertainty quantification reveals the importance of data variability and experimental design considerations for *in silico* proarrhythmia risk assessment. Front. Physiol. 8. 10.3389/fphys.2017.00917 29209226 PMC5702340

[B6] Christophe B. (2013). Simulation of early after-depolarisation in non-failing human ventricular myocytes: can this help cardiac safety pharmacology? Pharmacol. Rep. 65, 1281–1293. 10.1016/S1734-1140(13)71486-5 24399724

[B7] Christophe B. (2015). *In silico* study of transmural dispersion of repolarization in non-failing human ventricular myocytes: contribution to cardiac safety pharmacology. Br. J. Pharm. Res. 7, 88–101. 10.9734/BJPR/2015/17850

[B8] Colatsky T. Fermini B. Gintant G. Pierson J. B. Sager P. Sekino Y. (2016). The Comprehensive in Vitro Proarrhythmia Assay (CiPA) initiative—update on progress. J. Pharmacol. Toxicol. Methods 81, 15–20. 10.1016/j.vascn.2016.06.002 27282641

[B9] Costabal F. S. Matsuno K. Yao J. Perdikaris P. Kuhl E. (2019). Machine learning in drug development: characterizing the effect of 30 drugs on the QT interval using Gaussian process regression, sensitivity analysis, and uncertainty quantification. Comput. Methods Appl. Mech. Eng. 348, 313–333. 10.1016/j.cma.2019.01.033 PMC7454226 32863454

[B10] Costabal F. S. Yao J. Kuhl E. (2018). Predicting the cardiac toxicity of drugs using a novel multiscale exposure–response simulator. Comput. Methods Biomech. Biomed. Engin. 21, 232–246. 10.1080/10255842.2018.1439479 29493299 PMC6361171

[B11] Crumb W. J. Vicente J. Johannesen L. Strauss D. G. (2016). An evaluation of 30 clinical drugs against the Comprehensive in Vitro Proarrhythmia Assay (CiPA) proposed ion channel panel. J. Pharmacol. Toxicol. Methods 81, 251–262. 10.1016/j.vascn.2016.03.009 27060526

[B12] Cummins M. A. Dalal P. J. Bugana M. Severi S. Sobie E. A. (2014). Comprehensive analyses of ventricular myocyte models identify targets exhibiting favorable rate dependence. PLoS Comput. Biol. 10. 10.1371/journal.pcbi.1003543 PMC3967944 24675446

[B13] Devenyi R. A. Ortega F. A. Groenendaal W. Krogh-Madsen T. Christini D. J. Sobie E. A. (2017). Differential roles of two delayed rectifier potassium currents in regulation of ventricular action potential duration and arrhythmia susceptibility. J. Physiol. 595, 2301–2317. 10.1113/JP273191 27779762 PMC5374112

[B14] Devenyi R. A. Sobie E. A. (2016). There and back again: iterating between population-based modeling and experiments reveals surprising regulation of calcium transients in rat cardiac myocytes. J. Mol. Cell Cardiol. 96, 38–48. 10.1016/j.yjmcc.2015.07.016 26235057 PMC4733425

[B15] Dutta S. Chang K. C. Beattie K. A. Sheng J. Tran P. N. Wu W. W. (2017). Optimization of an *in silico* cardiac cell model for proarrhythmia risk assessment. Front. Physiol. 8. 10.3389/fphys.2017.00616 28878692 PMC5572155

[B16] Fermini B. Hancox J. C. Abi-Gerges N. Bridgland-Taylor M. Chaudhary K. W. Colatsky T. (2016). A new perspective in the field of cardiac safety testing through the comprehensive *in vitro* proarrhythmia assay paradigm, a new perspective in the field of cardiac safety testing through the comprehensive *in vitro* proarrhythmia assay paradigm. J. Biomol. Screen. 21, 1–11. 10.1177/1087057115594589 26170255

[B17] Gintant G. A. (2008). Preclinical torsades-de-pointes screens: advantages and limitations of surrogate and direct approaches in evaluating proarrhythmic risk. Pharmacol. Ther. 119, 199–209. 10.1016/j.pharmthera.2008.04.010 18621077

[B18] Herman J. Usher W. (2017). SALib: an open-source Python library for sensitivity analysis. J. Open Source Softw. 2. 10.21105/joss.00097

[B19] Homma T. Saltelli A. (1996). Importance measures in global sensitivity analysis of nonlinear models. Reliab. Eng. Syst. Safe 52, 1–17. 10.1016/0951-8320(96)00002-6

[B20] Hornberger G. M. U. O.V. Spear R. C. (1981). Approach to the preliminary analysis of environmental systems. J. Environ. Manage. (United States) 12, 1.

[B21] Iooss B. Lemaître P. (2014). A review on global sensitivity analysis methods. Boston, MA: Springer. 10.1007/978-1-4899-7547-8_5

[B22] January C. T. Riddle J. M. (1989). Early afterdepolarizations: mechanism of induction and block. A role for L-type Ca^2+^ current. Circ. Res. 64, 977–990. 10.1161/01.RES.64.5.977 2468430

[B23] Johnstone R. H. Bardenet R. Gavaghan D. J. Mirams G. R. (2016). Hierarchical Bayesian inference for ion channel screening dose-response data. Wellcome Open Res. 1, 6. 10.12688/wellcomeopenres.9945.2 27918599 PMC5134333

[B24] Kramer J. Obejero-Paz C. A. Myatt G. Kuryshev Y. A. Bruening-Wright A. Verducci J. S. (2013). MICE models: superior to the HERG model in predicting torsade de pointes. Sci. Rep. 3, 2100. 10.1038/srep02100 23812503 PMC3696896

[B25] Kubo T. Ashihara T. Tsubouchi T. Horie M. (2017). Significance of integrated *in silico* transmural ventricular wedge preparation models of human non-failing and failing hearts for safety evaluation of drug candidates. J. Pharmacol. Toxicol. Methods 83, 30–41. 10.1016/j.vascn.2016.08.007 27546811

[B26] Kucherenko S. Klymenko O. V. Shah N. (2017). Sobol’ indices for problems defined in non-rectangular domains. Reliab. Eng. Syst. Safe 167, 218–231. 10.1016/j.ress.2017.06.001

[B27] Lancaster M. C. Sobie E. A. (2016). Improved prediction of drug-induced torsades de pointes through simulations of dynamics and machine learning algorithms. Clin. Pharmacol. Ther. 100, 371–379. 10.1002/cpt.367 26950176 PMC6375298

[B28] Lee Y.-S. Liu O. Z. Hwang H. S. Knollmann B. C. Sobie E. A. (2013). Parameter sensitivity analysis of stochastic models provides insights into cardiac calcium sparks. Biophys. J. 104, 1142–1150. 10.1016/j.bpj.2012.12.055 23473497 PMC3870797

[B29] Li Z. Dutta S. Sheng J. Tran P. N. Wu W. Chang K. (2017). Improving the *in silico* assessment of proarrhythmia risk by combining hERG (human ether-à-go-go-related gene) channel-drug binding kinetics and multichannel pharmacology. Circ. Arrhythm. Electrophysiol. 10, e004628. 10.1161/CIRCEP.116.004628 28202629

[B30] Li Z. Ridder B. J. Han X. Wu W. W. Sheng J. Tran P. N. (2018). Assessment of an *in silico* mechanistic model for proarrhythmia risk prediction under the CiPA initiative. Clin. Pharmacol. Ther. 105 (2), 466–475. 10.1002/cpt.1184 30151907 PMC6492074

[B31] Loucks D. P. van Beek E. Stedinger J. R. Dijkman J. P. M. Villars M. T., (2017). Water resources systems planning and management: an introduction to methods, models and applications. Deltares: UNESCO-IHE, Springer. 10.1007/978-3-319-44234-1

[B32] McMillan B. Gavaghan D. J. Mirams G. R. (2017). Early afterdepolarisation tendency as a simulated pro-arrhythmic risk indicator. Toxicol. Res. 6, 912–921. 10.1039/C7TX00141J PMC5779076 29456831

[B33] Milnes J. T. Witchel H. J. Leaney J. L. Leishman D. J. Hancox J. C. (2010). Investigating dynamic protocol-dependence of hERG potassium channel inhibition at 37 degrees C: cisapride versus dofetilide. J. Pharmacol. Toxicol. Methods 61, 178–191. 10.1016/j.vascn.2010.02.007 20172036

[B34] Mirams G. R. Cui Y. Sher A. Fink M. Cooper J. Heath B. M. (2011). Simulation of multiple ion channel block provides improved early prediction of compounds’ clinical torsadogenic risk. Cardiovasc. Res. 91, 53–61. 10.1093/cvr/cvr044 21300721 PMC3112019

[B35] Mirams G. R. Pathmanathan P. Gray R. A. Challenor P. Clayton R. H. (2016). Uncertainty and variability in computational and mathematical models of cardiac physiology. J. Physiol. 594, 6833–6847. 10.1113/JP271671 26990229 PMC5134370

[B36] Mistry H. B. (2018). Complex versus simple models: ion-channel cardiac toxicity prediction. PeerJ 6, e4352 10.7717/peerj.4352 29423349 PMC5804316

[B37] Mistry H. B. Davies M. R. DiVeroli G. Y. (2015). A new classifier-based strategy for in-silico ion-channel cardiac drug safety assessment. Front. Pharmacol. 6. 10.3389/fphar.2015.00059 25852560 PMC4371651

[B38] Morotti S. Grandi E. (2016). Logistic regression analysis of populations of electrophysiological models to assess proarrythmic risk. MethodsX 4, 25–34. 10.1016/j.mex.2016.12.002 28116246 PMC5225282

[B39] O’Hara T. Virág L. Varró A. Rudy Y. (2011). Simulation of the undiseased human cardiac ventricular action potential: model formulation and experimental validation. PLoS Comput. Biol. 7, e1002061. 10.1371/journal.pcbi.1002061 21637795 PMC3102752

[B40] Okada J.-I. Yoshinaga T. Kurokawa J. Washio T. Furukawa T. Sawada K. (2015). Screening system for drug-induced arrhythmogenic risk combining a patch clamp and heart simulator. Sci. Adv. 1, e1400142. 10.1126/sciadv.1400142 26601174 PMC4640654

[B41] Parikh J. DiAchille P. Kozloski J. Gurev V. (2019). Intrinsic structure of model-derived metrics for *in silico* proarrhytmic risk assessment identified by global sensitivity analysis. bioRxiv. 10.1101/543926

[B42] Parikh J. Gurev V. Rice J. J. (2017). Novel two-step classifier for torsades de pointes risk stratification from direct features. Front. Pharmacol. 8. 10.3389/fphar.2017.00816 29184497 PMC5694470

[B43] Passini E. Britton O. J. Lu H. R. Rohrbacher J. Hermans A. N. Gallacher D. J. (2017). Human *in silico* drug trials demonstrate higher accuracy than animal models in predicting clinical pro-arrhythmic cardiotoxicity. Front. Physiol. 8, 668. 10.3389/fphys.2017.00668 28955244 PMC5601077

[B44] Pedregosa F. Varoquaux G. Gramfort A. Michel V. Thirion B. Grisel O. (2011). Scikit-Learn: machine learning in Python. J. Mach. Learn. Res. 12, 2825–2830.

[B45] Pianosi F. Beven K. Freer J. Hall J. W. Rougier J. Stephenson D. B. (2016). Sensitivity analysis of environmental models: a systematic review with practical workflow. Environ. Model Softw. 79, 214–232. 10.1016/j.envsoft.2016.02.008

[B46] Redfern W. S. Carlsson L. Davis A. S. Lynch W. G. MacKenzie I. Palethorpe S. (2003). Relationships between preclinical cardiac electrophysiology, clinical QT interval prolongation and torsade de pointes for a broad range of drugs: evidence for a provisional safety margin in drug development. Cardiovasc. Res. 58, 32–45. 10.1016/S0008-6363(02)00846-5 12667944

[B47] Romero L. Pueyo E. Fink M. Rodríguez B. (2009). Impact of ionic current variability on human ventricular cellular electrophysiology. Am. J. Physiol. Heart Circ. Physiol. 297, H1436–H1445. 10.1152/ajpheart.00263.2009 19648254

[B48] Sadrieh A. Mann S. A. Subbiah R. N. Domanski L. Taylor J. A. Vandenberg J. I. (2013). Quantifying the origins of population variability in cardiac electrical activity through sensitivity analysis of the electrocardiogram. J. Physiol. 591, 4207–4222. 10.1113/jphysiol.2013.251710 23551947 PMC3779112

[B49] Sager P. T. Gintant G. Turner J. R. Pettit S. Stockbridge N. (2014). Rechanneling the cardiac proarrhythmia safety paradigm: a meeting report from the Cardiac Safety Research Consortium. Am. Heart J. 167, 292–300. 10.1016/j.ahj.2013.11.004 24576511

[B50] Saltelli A. (2002). Making best use of model evaluations to compute sensitivity indices. Comput. Phys. Commun. 145, 280–297. 10.1016/S0010-4655(02)00280-1

[B51] Saltelli A. Ratto M. Andres T. Campolongo F. Cariboni J. Gatelli D. et al. (2008). Global sensitivity analysis: the primer. (Chichester: John Wiley & Sons). 10.1002/9780470725184

[B52] Sarkar A. X. Sobie E. A. (2010). Regression analysis for constraining free parameters in electrophysiological models of cardiac cells. PLoS Comput. Biol. 6 (9), e1000914. 10.1371/journal.pcbi.1000914 20824123 PMC2932676

[B53] Shimizu W. Antzelevitch C. (1998). Cellular basis for the ECG features of the LQT1 form of the long-QT syndrome: effects of beta-adrenergic agonists and antagonists and sodium channel blockers on transmural dispersion of repolarization and torsade de pointes. Circulation 98, 2314–2322. 10.1161/01.CIR.98.21.2314 9826320

[B54] Sobie E. A. (2009). Parameter sensitivity analysis in electrophysiological models using multivariable regression. Biophys. J. 96, 1264–1274. 10.1016/j.bpj.2008.10.056 19217846 PMC2717232

[B55] Sobol’ I. M. (2001). Global sensitivity indices for nonlinear mathematical models and their Monte Carlo estimates. Math Comput. Simul. 55, 271–280. 10.1016/S0378-4754(00)00270-6

[B56] Trenor B. Gomis-Tena J. Cardona K. Romero L. Rajamani S. Belardinelli L. (2013). *In silico* assessment of drug safety in human heart applied to late sodium current blockers. Channels (Austin, Tex.) 7, 249–262. 10.4161/chan.24905 PMC3989354 23696033

[B57] Veroli G. Y. D. Davies M. R. Zhang H. Abi-Gerges N. Boyett M. R. (2014). hERG inhibitors with similar potency but different binding kinetics do not pose the same proarrhythmic risk: implications for drug safety assessment. J. Cardiovasc. Electrophysiol. 25, 197–207. 10.1111/jce.12289 24118558

[B58] Weiss J. N. Garfinkel A. Karagueuzian H. S. Chen P.-S. Qu Z. (2010). Early afterdepolarizations and cardiac arrhythmias. Heart Rhythm 7, 1891–1899. 10.1016/j.hrthm.2010.09.017 20868774 PMC3005298

[B59] Yan G. X. Wu Y. Liu T. Wang J. Marinchak R. A. Kowey P. R. (2001). Phase 2 early afterdepolarization as a trigger of polymorphic ventricular tachycardia in acquired long-QT syndrome: direct evidence from intracellular recordings in the intact left ventricular wall. Circulation 103, 2851–2856. 10.1161/01.CIR.103.23.2851 11401944

[B60] Yap Y. G. Camm A. J. (2003). Drug induced QT prolongation and torsades de pointes. Heart 89, 1363–1372. 10.1136/heart.89.11.1363 14594906 PMC1767957

[B61] Zeng J. Rudy Y. (1995). Early afterdepolarizations in cardiac myocytes: mechanism and rate dependence. Biophys. J. 68, 949–964. 10.1016/S0006-3495(95)80271-7 7538806 PMC1281819

