# Supplementary material for: Global Sensitivity Analysis of Ventricular Myocyte Model-Derived Metrics for Proarrhythmic Risk Assessment
Source: Front Pharmacol. 2019 Oct 2;10:1054. doi: 10.3389/fphar.2019.01054 (PMC6797832; doi:10.3389/fphar.2019.01054)
Supplement: Supplementary file 1 [file DataSheet_1.pdf]

Supplementary Material

1 SUPPLEMENTARY DATA

1.1 CiPA training and validations compounds dataset

IC_{50} values and Hill coefficients describing the effects of the CiPA training drugs on non-hERG channels were extracted from Li et al. (2017). Similarly, median values from the manual patch clamp experiments on the CiPA validation drugs were taken from Li et al. (2018). The values are reported in Table S1.

Table S1. IC_{50} values (nM) and Hill coefficients for non-hERG channels

	ICaL_IC50	ICaL_h	IK1_IC50	IK1_h	IKs_IC50	IKs_h	INa_IC50	INa_h	INaL_IC50	INaL_h	Ito_IC50	Ito_h	hERG_IC50	hERG_h
astemizole	553.0	1.2000	-	-	-	-	5.410000e+03	0.7600	10300.0	2.3000	-	-	-	-
azimilide	13200.0	0.7100	-	-	-	-	3.630000e+05	0.7200	2940000.0	0.4700	-	-	-	-
bepridil	2808.0	0.6486	-	-	28630.0	0.7061	2.929000e+03	1.1640	1814.0	1.4160	8.594000e+03	3.5410	51.340	0.9293
chlorpromazine	8192.0	0.8441	9270.0	0.6878	-	-	4.536000e+03	1.9950	4560.0	0.9379	1.762000e+07	0.3654	975.200	0.8281
cisapride	9267000.0	0.4261	29480.0	0.5133	81170000.0	0.2921	-	-	-	-	2.191000e+05	0.2430	11.270	0.6210
clarithromycin	38100.0	0.8800	-	-	-	-	1.090000e+06	0.8900	1810000.0	3.0000	-	-	-	-
clozapine	5490.0	0.9400	-	-	-	-	2.570000e+05	0.5900	73600.0	2.0000	-	-	-	-
diltiazem	112.1	0.7142	-	-	-	-	1.109000e+05	0.7022	21870.0	0.6779	2.822000e+09	0.1696	13140.000	0.9119
disopyramide	32900.0	0.6900	-	-	-	-	1.920000e+05	1.3000	377000.0	2.1000	-	-	-	-
dofetilide	260.3	1.1630	394.4	0.7650	-	-	3.805000e+02	0.8920	753100.0	0.2597	1.882000e+01	0.7712	6.125	1.0790
domperidone	73.6	0.4900	-	-	-	-	4.190000e+04	1.5000	225000.0	2.1000	-	-	-	-
droperidol	3230.0	1.2000	-	-	-	-	3.660000e+04	2.5000	33800.0	2.8000	-	-	-	-
ibutilide	37000.0	0.8600	-	-	-	-	2.410000e+04	2.3000	287000.0	2.4000	-	-	-	-
loratadine	703.0	0.5600	-	-	-	-	1.130000e+05	1.4000	192000.0	2.1000	-	-	-	-
metoprolol	3280000.0	0.5400	-	-	-	-	3.030000e+04	0.6100	630000.0	0.6600	-	-	-	-
mexiletine	38240.0	1.0310	-	-	-	-	-	-	8957.0	1.4090	-	-	29070.000	0.8928
nifedipine	11.4	0.6700	-	-	-	-	2.760000e+04	1.1000	45600.0	4.5000	-	-	-	-
nitrendipine	35.7	0.5000	-	-	-	-	2.240000e+04	0.5800	70700.0	3.2000	-	-	-	-
ondansetron	22550.0	0.7526	-	-	569800.0	0.6535	5.767000e+04	1.0200	19180.0	1.0350	1.023000e+06	0.9891	1325.000	0.9210
pimozide	64.5	0.4500	-	-	-	-	1.020000e+04	0.4600	1910.0	1.9100	-	-	-	-
quinidine	51590.0	0.5892	39590000.0	0.3468	4899.0	1.3630	1.233000e+04	1.4940	9417.0	1.3370	3.487000e+03	1.2820	986.300	0.8404
ranolazine	-	-	-	-	36160000.0	0.5191	6.877000e+04	1.4250	7884.0	0.9450	-	-	8208.000	0.8576
risperidone	1470.0	0.5900	-	-	-	-	5.340000e+05	0.7100	11400000.0	5.8000	-	-	-	-
sotalol	7062000.0	0.8651	3050000.0	1.2040	4222000.0	1.1670	1.144000e+09	0.5089	-	-	4.314000e+07	0.6632	107100.000	0.7850
tamoxifen	5720.0	0.7600	-	-	-	-	8.400000e+04	0.8200	3640000.0	4.0000	-	-	-	-
terfenadine	700.4	0.6601	-	-	399800.0	0.5430	4.803000e+03	1.0150	20060.0	0.6011	2.400000e+05	0.2559	20.400	0.6118
vandetanib	6060.0	0.7200	-	-	-	-	8.090000e+04	1.9000	3790000.0	0.8200	-	-	-	-
verapamil	201.8	1.0970	348800000.0	0.2728	-	-	-	-	7028.0	1.0310	1.343000e+04	0.8222	295.600	0.9378

hERG dynamic data was extracted from Li et al. (2017) and Li et al. (2018) for the training and validation compounds, respectively, as listed in Table S2. In the simulations, the reported median values of parameters were used.

Table S2. Drug-hERG binding dynamic parameters.

	$Kmax$	Ku	n	$halfmax$	$Vhalf$
astemizole	2.420	0.000033	1.4490	4.883000e+00	-6.110
azimilide	654000.000	0.008250	0.6028	1.413000e+07	-8.821
bepridil	5594000.000	0.000172	0.9374	1.472000e+08	-61.340
chlorpromazine	157900.000	0.046710	0.8871	4.351000e+07	-14.450
cisapride	10.220	0.000416	0.9615	4.232000e+01	-167.400
clarithromycin	92.890	0.012160	0.7867	2.229000e+05	-102.800
clozapine	7.486	0.029890	1.3670	9.048000e+04	-8.810
diltiazem	182500.000	0.282000	0.9382	6.677000e+08	-90.650
disopyramide	3.685	0.121600	0.7894	4.473000e+04	-78.110
dofetilide	35.100	0.000018	1.0800	2.166000e+02	-1.000
domperidone	3.339	0.000356	0.7026	1.609000e+01	-65.650
droperidol	14.210	0.001256	0.5780	1.165000e+02	-78.680
ibutilide	14.570	0.000061	0.9231	3.863000e+01	-9.771
loratadine	376500.000	0.009642	0.8368	4.770000e+08	-1.000
metoprolol	31790.000	0.850800	0.8110	4.223000e+07	-89.350
mexiletine	15.000	0.071140	1.1390	7.230000e+05	-87.510
nifedipine	4.748	0.991600	1.2350	9.752000e+08	-87.370
nitrendipine	1.713	0.981900	1.9230	5.669000e+08	-61.580
ondansetron	172000.000	0.023240	0.8910	5.224000e+07	-82.200
pimozide	10.070	0.000046	0.8714	5.601000e+00	-158.500
quinidine	275.700	0.004103	0.8488	5.383000e+04	-61.350
ranolazine	52.840	0.020350	0.9532	1.430000e+05	-94.990
risperidone	3.930	0.001151	1.1220	7.528000e+02	-80.430
sotalol	96190.000	0.022250	0.7513	3.856000e+08	-51.500
tamoxifen	3.900	0.011750	2.0000	4.067000e+05	-2.036
terfenadine	102200.000	0.000078	0.6502	4.095000e+05	-81.630
vandetanib	36.280	0.019740	0.7126	2.223000e+03	-48.550
verapamil	1694000.000	0.000816	1.0430	3.356000e+08	-97.080

The known TdP categories and maximum effective free therapeutic concentrations for the validation drugs (Li et al. (2017, 2018)) are listed in Table S3.

Table S3. Drug Therapeutic concentrations and risk

Drug	EFTPC (nM)	Risk
astemizole	0.260	Medium
azimilide	70.000	High
bepridil	33.000	High
chlorpromazine	38.000	Medium
cisapride	2.600	Medium
clarithromycin	1206.000	Medium
clozapine	71.000	Medium
diltiazem	122.000	Low
disopyramide	742.000	High
dofetilide	2.000	High
domperidone	19.000	Medium
droperidol	6.330	Medium
ibutilide	140.000	High
loratadine	0.450	Low
metoprolol	1800.000	Low
mexiletine	4129.000	Low
nifedipine	7.700	Low
nitrendipine	3.020	Low
ondansetron	139.000	Medium
pimozide	0.431	Medium
quinidine	3237.000	High
ranolazine	1948.200	Low
risperidone	1.810	Medium
sotalol	14690.000	High
tamoxifen	21.000	Low
terfenadine	4.000	Medium
vandetanib	255.400	High
verapamil	81.000	Low

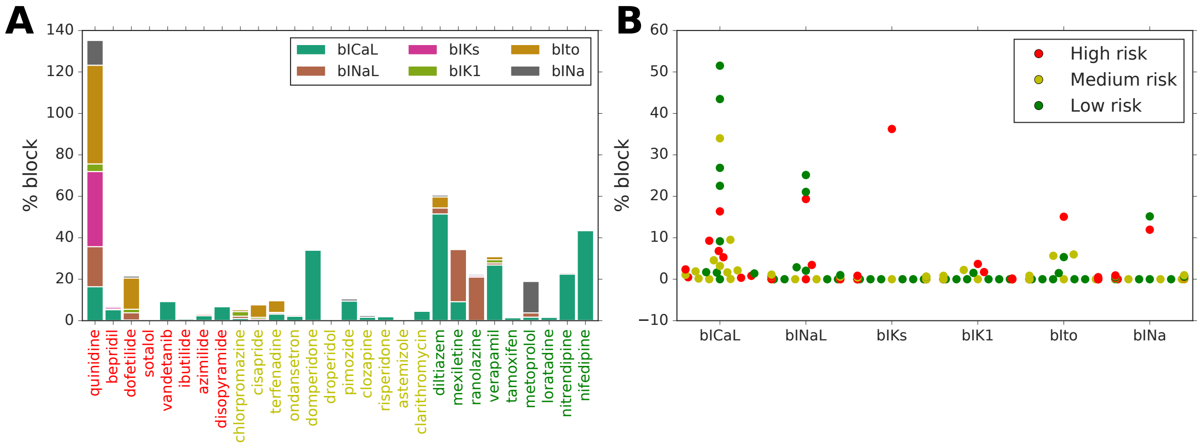

Figure S1. Drug-induced block of non-hERG ion channels for 28 CiPA compounds at their EFTPC based on measurements from an *in vitro* assay Crumb et al. (2016); Li et al. (2018). **A**: Stacked bar chart of six ion channel current block values for the 28 drugs. **B**: A swarm plot of block values of six ion channel currents categorized into high, medium, and low risk groups.

1.2 Sampling schemes

1.2.1 Saltelli sampling scheme

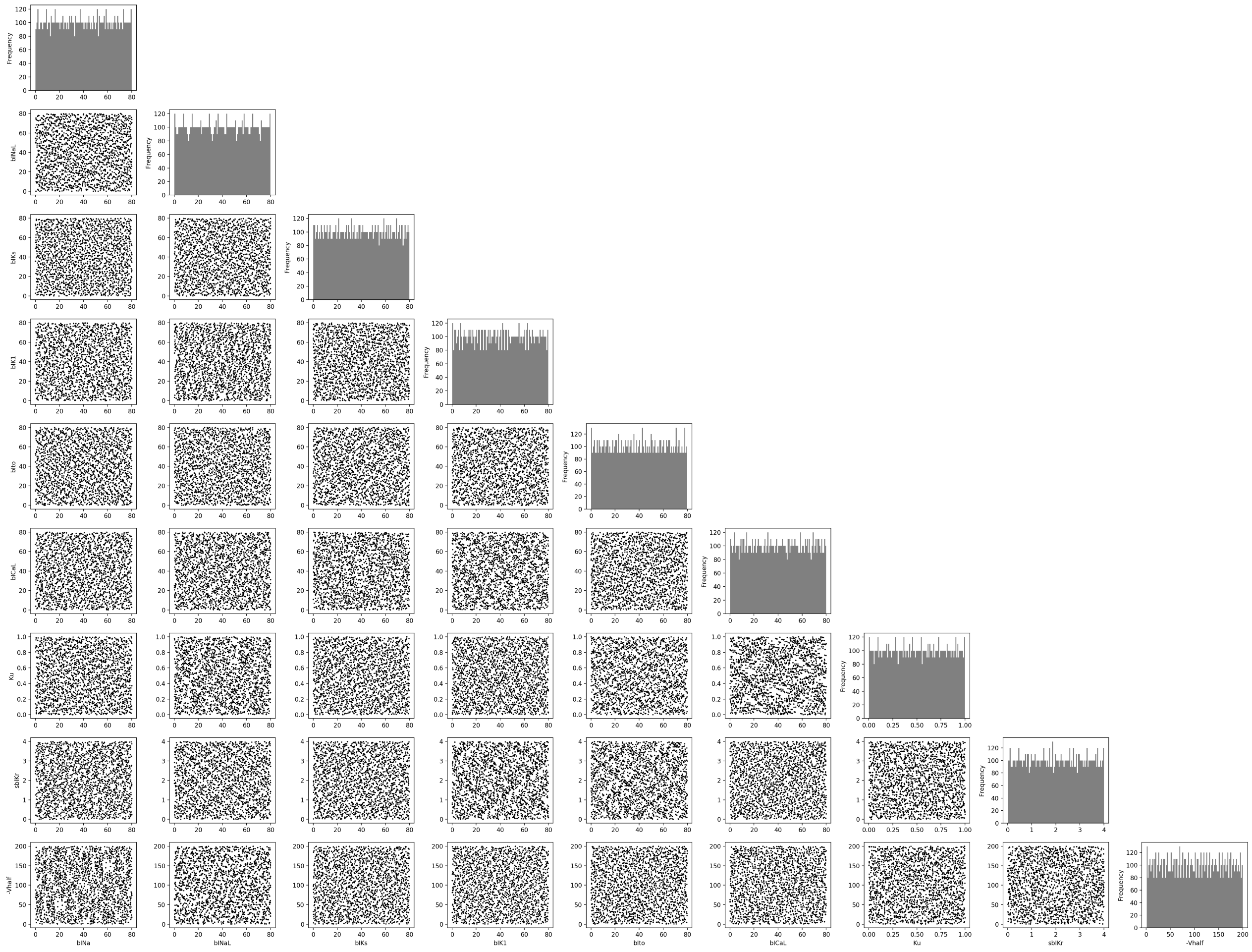

Figure S2. Marginal and joint distributions of the 9 input parameters for the 10000 virtual drugs (Virtual Drug Population I) uniformly sampled via Saltelli's scheme.

1.2.2 Sampling drugs using distribution of parameters of 28 CiPA drugs as a prior

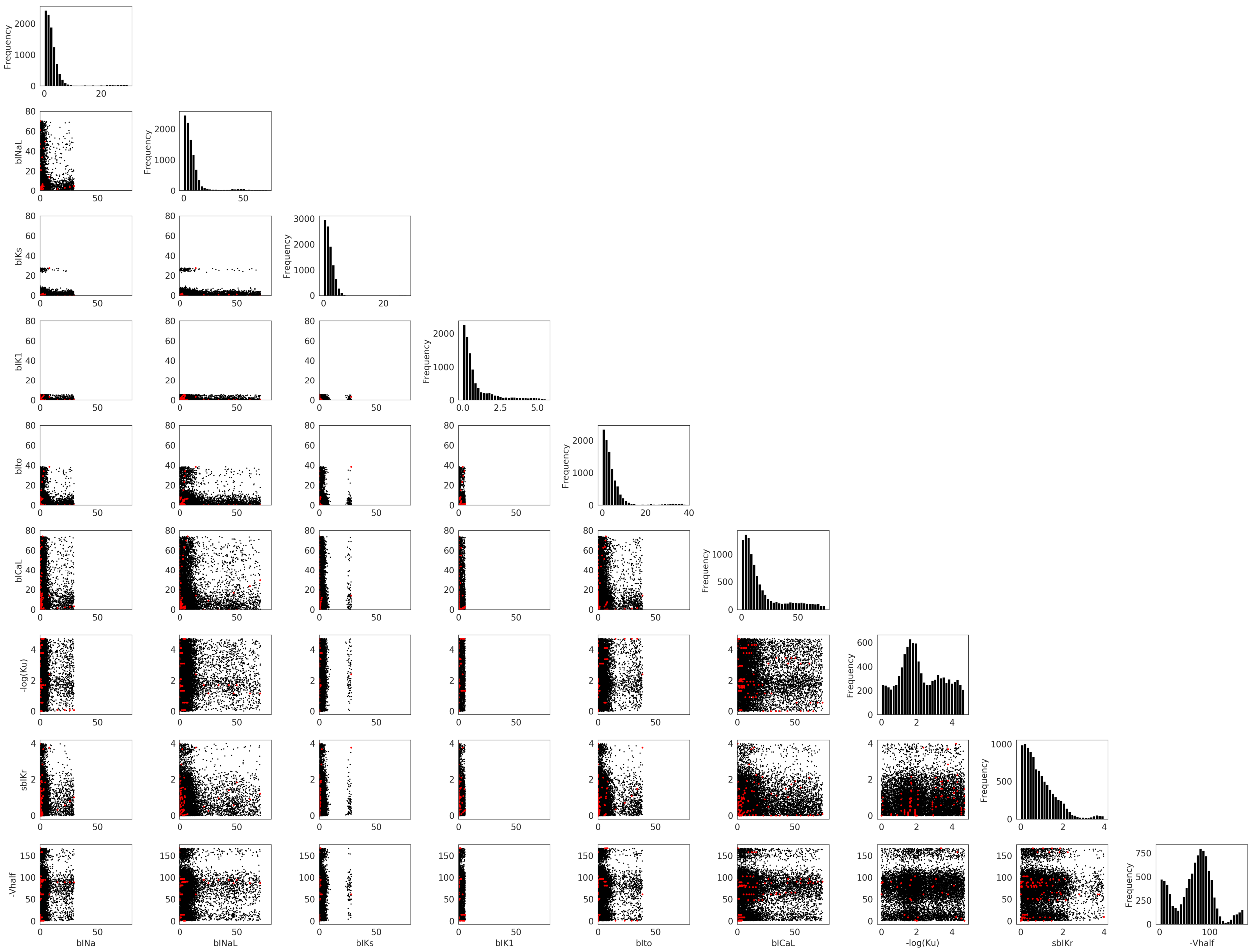

Figure S3. Marginal and joint distributions of the 9 input parameters corresponding for the 10000 virtual drugs (Virtual Drug Population II) sampled from the prior parameter distribution of the "CiPA drugs" (at 1-4x EFPTC) obtained via kernel density estimation method. The red dots in the joint distribution plot represent parameter values for the 28 CiPA drugs at 1-4x EFTPC.

1.3 Sobol sensitivity analysis

1.3.1 Simple examples highlighting differences between in sensitivity estimates computed via Sobol sensitivity and multivariate linear regression methods

Multivariate linear regression has been used in the past Sobie (2009) to identify sensitivity of outputs from cardiac cell models to changes in input parameters. To illustrate the differences between linear regression [1] and variance-based sensitivity analysis, in the Figure S4, we provide few examples highlighting the differences between the variance based sensitivity measures and sensitivity coefficients from the linear regression. For a hypothetical output feature (Feature1 in S4 A) that can be perfectly fitted by a linear regression of model input parameters ($Feature1 = 1.5P_1 + P_2 + 5$) the sensitivity coefficients obtained using the two methods are identical (Figure S4). In contrast, the sensitivity estimates for model features that present nonlinear input-output relationships are inaccurate when using the linear regression methods, and the variance-based analysis provides better estimates for such situations. The metric $S1$ captures the contribution of the first order as well as all higher order terms for the individual input. For the $Feature2$ in the Figure S4 the $S1$ term captures the contribution of both P_1 and P_1^2 terms. The metric $S2$ captures all the second order interaction terms (i.e., P_1P_2). The variance in the hypothetical $Feature3$ in the Figure S4 depends on the interaction between $P1$ and $P2$ parameters, which is captured by the $S2$ index, and also in the total sensitivity index ST, which includes all higher-order interaction terms, including $S2$. The $S2$ index of 0.38 indicates a contribution of 38% in the variance of $Feature3$ from the second-order interaction term (Figure S4 B). Hence, the variance based sensitivity analysis provides a more general method, which allows us to estimate the contributions of parameter interactions and nonlinear effects on regulation of the output features.

In addition we also calculated global sensitivity estimates using Mean Decrease Accuracy (MDA) method, which provides clear interpretation of feature ranking. We first approximate the derived metric by a metamodel (i.e., random forest regressor model for the particular case) Then, we evaluated the accuracy of the metarepresentation upon random permutations of the values of a given feature. Losses in accuracy measured for each of the permutations provided us with global sensitivity estimates. We observed that the sensitivity estimates provided by MDA were similar to the Sobol ST index.

[1] Here and further in the paper, we discuss linear regressions with input features typically used in the sensitivity analysis, i.e., regressions without any nonlinear features constructed from the input parameters.

A

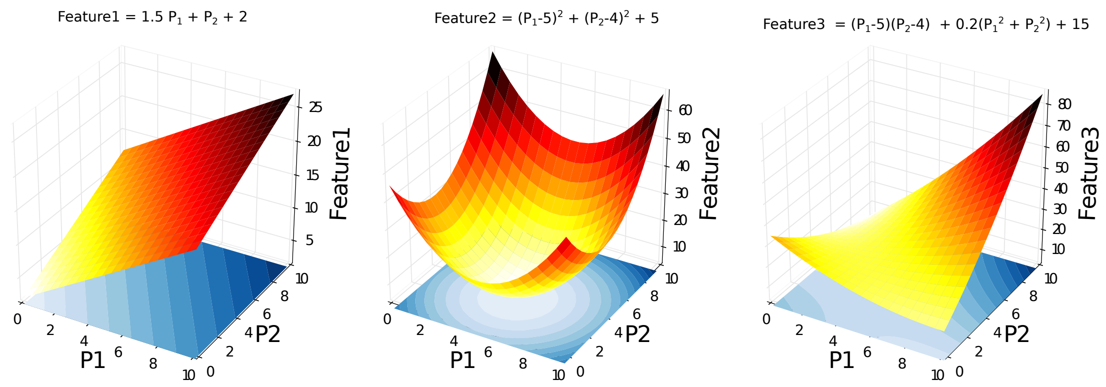

B

	**Feature1**		**Feature2**		**Feature3**	
	P1	*P2*	*P1*	*P2*	*P1*	*P2*
Method 1: Linear Regression						
LinR	0.69	0.31	0.00	0.23	0.41	0.18
Method 2: Sobol Sensitvity Analysis						
S1	0.69	0.31	0.38	0.62	0.42	0.20
S2	0.0		0.0		0.38	
ST	0.69	0.31	0.38	0.62	0.80	0.58
STi/ΣSTi	0.69	0.31	0.38	0.62	0.58	0.42
Method 3: Mean decrease in accuracy (Random Forest)						
$MDA_i/\Sigma MDA_i$	0.69	0.31	0.39	0.61	0.58	0.42

Figure S4. Example highlighting the difference between the multivariate linear regression and variance-based sensitivity methods. **A:** Schematic of variation in three synthetic features due to variation in two input parameters. **B:** Sensitivity estimates of the three synthetic features from **A** using multivariate linear regression, variance-based sensitivity method and MDA methods.

1.3.2 Sobol sensitivity indices

The manuscript reports first-order ($S1$) and total sensitivity indices (ST) for the $qNet$, $APD90$ and $peakCa$ output metrics extracted from the endo cell model. Figures S5 show the estimated values of the Sobol indices ($S1$ and ST) for six additional derived features ($APD50$, $diastolicCa$, $CaTD50$, $CaTD90$, and $peakV_m$).

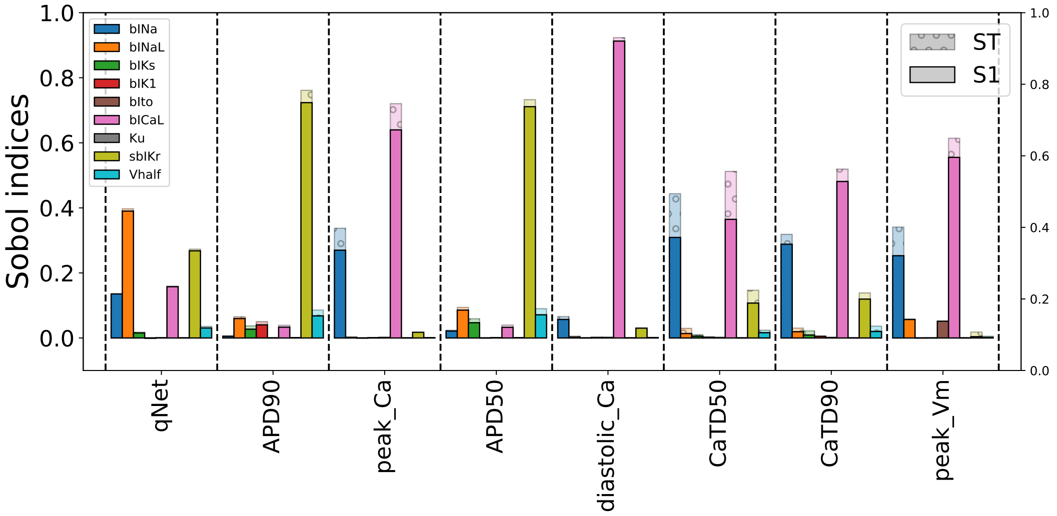

Figure S5. Plot of estimated first-order and total Sobol sensitivity indices (S1 and ST) to evaluate relative contributions of different input parameters on output variablity of 9 model-derived metrics including $qNet$, $APD90$ and $peakCa$ (endo cell model)

1.3.3 Sobol sensitivity indices vs. sensitivity indices obtained from multivariate linear regression

The link between GSA and feature selection is well illustrated in the following example. We consider that some model-derived metric M can be well approximated by a multivariatie quadratic polynomial, with direct features as input variables of the polynomial. The equation of the polynomial is

$$M = c + \sum_i a_i x_i + \sum_{ij} b_{ij} x_i x_j, \tag{S1}$$

where $\{x_i\}$ is a vector of direct features; $\{a_i\}$ and $\{b_{ij}\}$ are a vector and a symmetric matrix of coefficients for the linear and quadratic terms of the poylnomial, respectively, and c is a constant. The components of $\{x_i\}$, i.e., the individual direct features, are assumed to be uniformly distributed with expectation e_i and variance v_i. In this case, the effect of each direct feature x_i and the joint effect of direct features $x_i x_j$ on variance in M are given by

$$V_i = (a_i + \sum_{\substack{k \\ k \neq i}} 2b_{ik} e_i)^2 v_i + b_{ii}^2 var(x_i^2) + 2b_{ii}(a_i + \sum_{\substack{k \\ k \neq i}} 2b_{ik} e_i) cov(x_i, x_i^2), \tag{S2}$$

$$V_{ij} = 4b_{ij}^2 \left(v_i v_j + e_j^2 v_i + v_j e_i^2 - cov(x_i x_j, x_i) e_j - cov(x_j x_i, x_j) e_i \right). \tag{S3}$$

Typically, to rank the features based on their importance, feature scaling is carried out on the inputs before performing the regression. If each of the uniformly distributed direct features x_i are centered and normalized to have $e_i = 0$ and $v_i = 1$, then

$$V_i = a_i^2 + \frac{4}{5} b_{ii}^2, \tag{S4}$$

$$V_{ij} = 4b_{ij}^2. \tag{S5}$$

Hence, the first- and second-order Sobol indices can be represented as

$$S1_i = \frac{a_i^2 + \frac{4}{5} b_{ii}^2}{var(M)}, \tag{S6}$$

$$S2_{ij} = \frac{4b_{ij}^2}{var(M)}. \tag{S7}$$

Expressions in (S6)-(S7) provide a link between calculated variances in the Sobol sensitivity method and the coefficients of the polynomial. Indeed, to demonstrate these relationships, we fitted a linear regression to approximate model-derived metrics ($APD90$, $qNet$, and $peakCa$) accounting for only the first-order effects of the direct features, i.e., $M = c + \sum a_i x_i$. Then, the squared coefficients of the regression are normalized by the variance of derived features and compared with Sobol $S1$ index. As expected, in the presence of minor interactions and nonlinear effects for these derived metrics, we observe a good match between indices estimated from the regression coefficients and the Sobol $S1$ indices as shown in Figure S6, even when considering only the first-order effects to construct the regression.

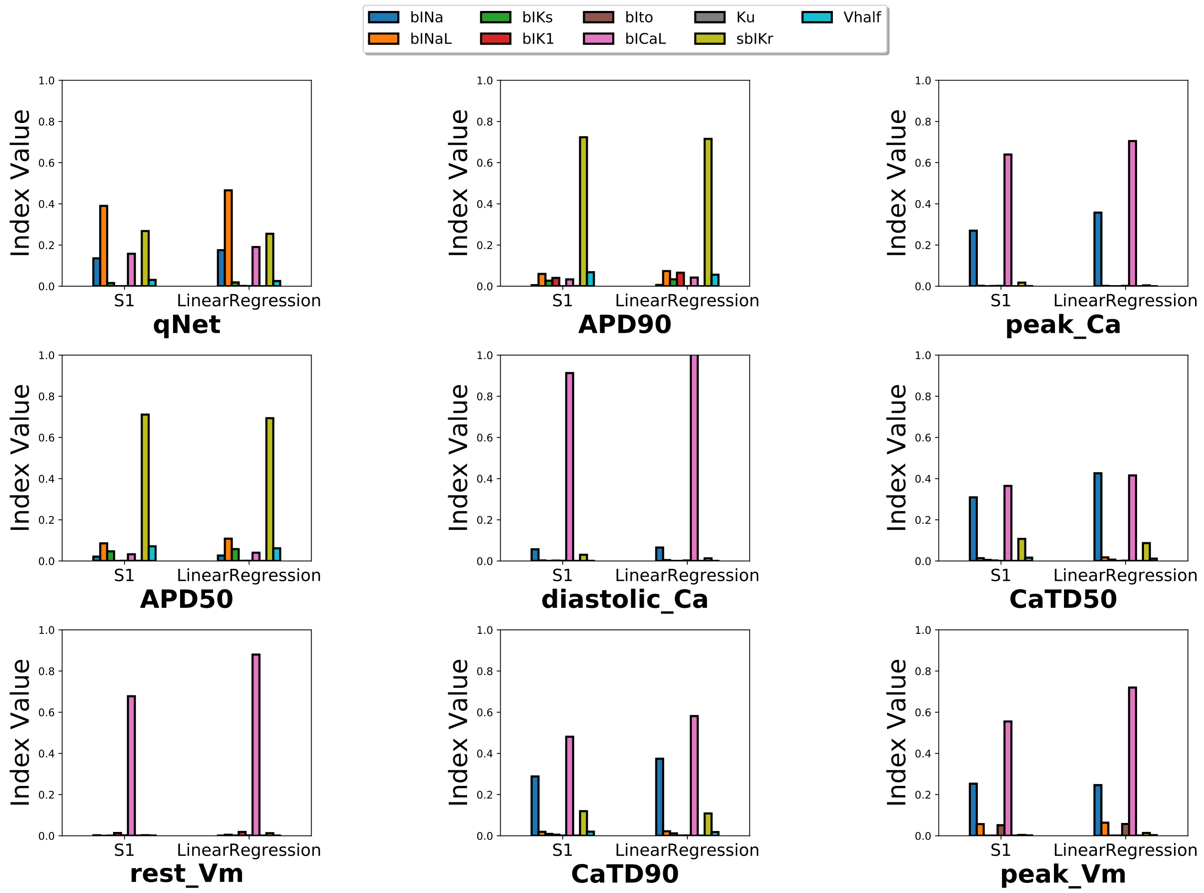

Figure S6. Comparison of sensitivity index estimated via multivariate linear regression and Sobol first-order index $S1$ for $qNet$, $APD90$, $peakCa$, $APD50$, $diastolicCa$, $CaTD50$, $restVm$, $CaTD90$ and $peakVm$.

1.4 Correlations between model-derived metrics for the 28 "CiPA drugs"

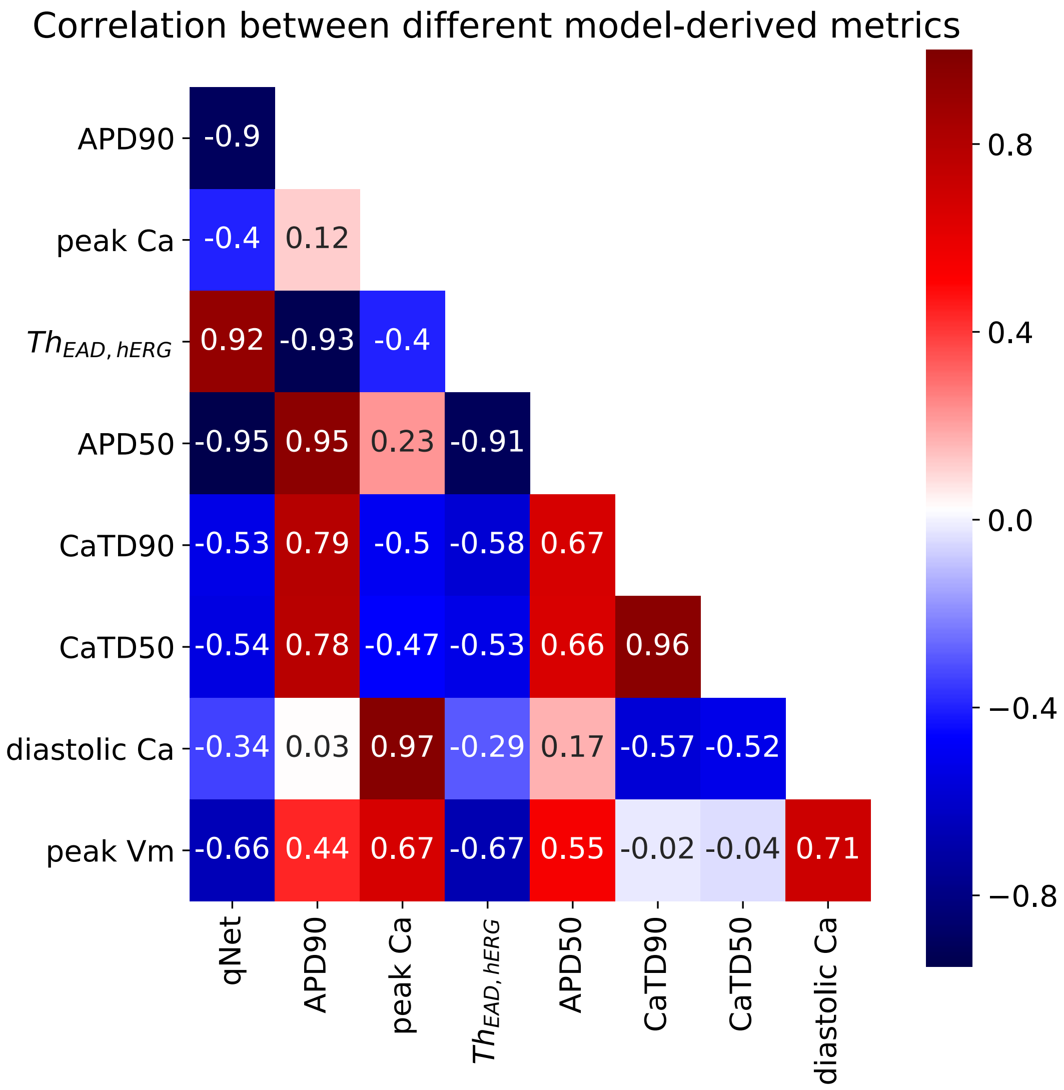

Figure S7. Heatmap of correlation between different model-derived metrics.

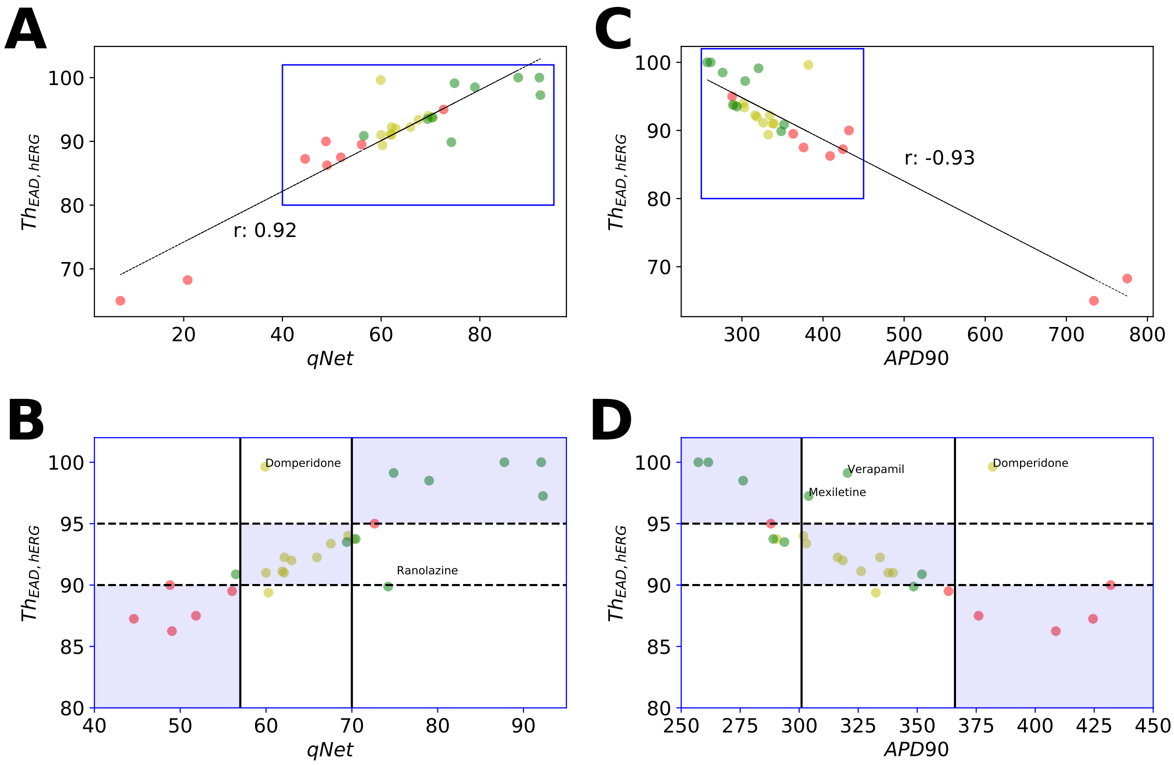

Figure S8. Scatter plot of $qNet$ vs $Th_{EAD,hERG}$ metrics. **A:** For all the 28 "CiPA drugs" a high correlation of 0.92 was observed. A region of interest is expanded in **B:** to show details of separation among the drugs across the independently determined ranges for low, intermediate and high risk based on $qNet$ (solid black lines) and $Th_{EAD,hERG}$ (dotted black lines) metrics. Scatter plot of $APD90$ vs $Th_{EAD,hERG}$ metrics. **C:** For all the 28 "CiPA drugs" a high correlation of -0.93 was observed. A region of interest is expanded in **D:** to show details of separation among the drugs across the independently determined ranges for low, intermediate and high risk based on $APD90$ (solid black lines) and $Th_{EAD,hERG}$ (dotted black lines) metrics. Blue regions show where both the $qNet$ and $Th_{EAD,hERG}$ metric agree. The high, intermediate and low risk drugs are colored in red, yellow and green, respectively, based on their torsadogenic risk. See the Supplemental Material for an additional plot of $APD90$ vs $Th_{EAD,hERG}$ metrics.

1.5 Evaluation of most influential parameters allowing accurate risk discrimination of virtual drugs using Monte Carlo filtering

1.5.1 Estimated thresholds via logistic regression on classifying the CiPA drugs into low, intermediate and high risk groups

Table S4. Thresholds for tertiary classification of 28 CiPA drugs

$APD90$		$Th_{EAD,hERG}$		$qNet$	
High-Intermediate	Intermediate-Low	High-Intermediate	Intermediate-Low	High-Intermediate	Intermediate-Low
307	377	90	95	57	70

1.5.2 Cumulative distribution functions estimated for classification of virtual drugs into low, intermediate and high risk

Table S5. Total number of virtual drugs observed in low, intermediate and high risk subsets on classification using $qNet$ and $Th_{EAD,hERG}$ metric.

Sampling scheme	Metric	Total	Low risk	Medium risk	High risk
S1	$qNet$	10000	3872	2099	4029
S2	$qNet$	10000	3390	4063	2547
S1	$Th_{EAD,hERG}$	10000	3580	1406	5014
S2	$Th_{EAD,hERG}$	10000	2901	4439	2660

S1: Almost uniform sampling across the parametric space using Saltelli sampling scheme (Figure S2)
S2: Non uniform sampling across the parametric space based on prior distribution of parameters from CiPA drug measurements (Figure S3)

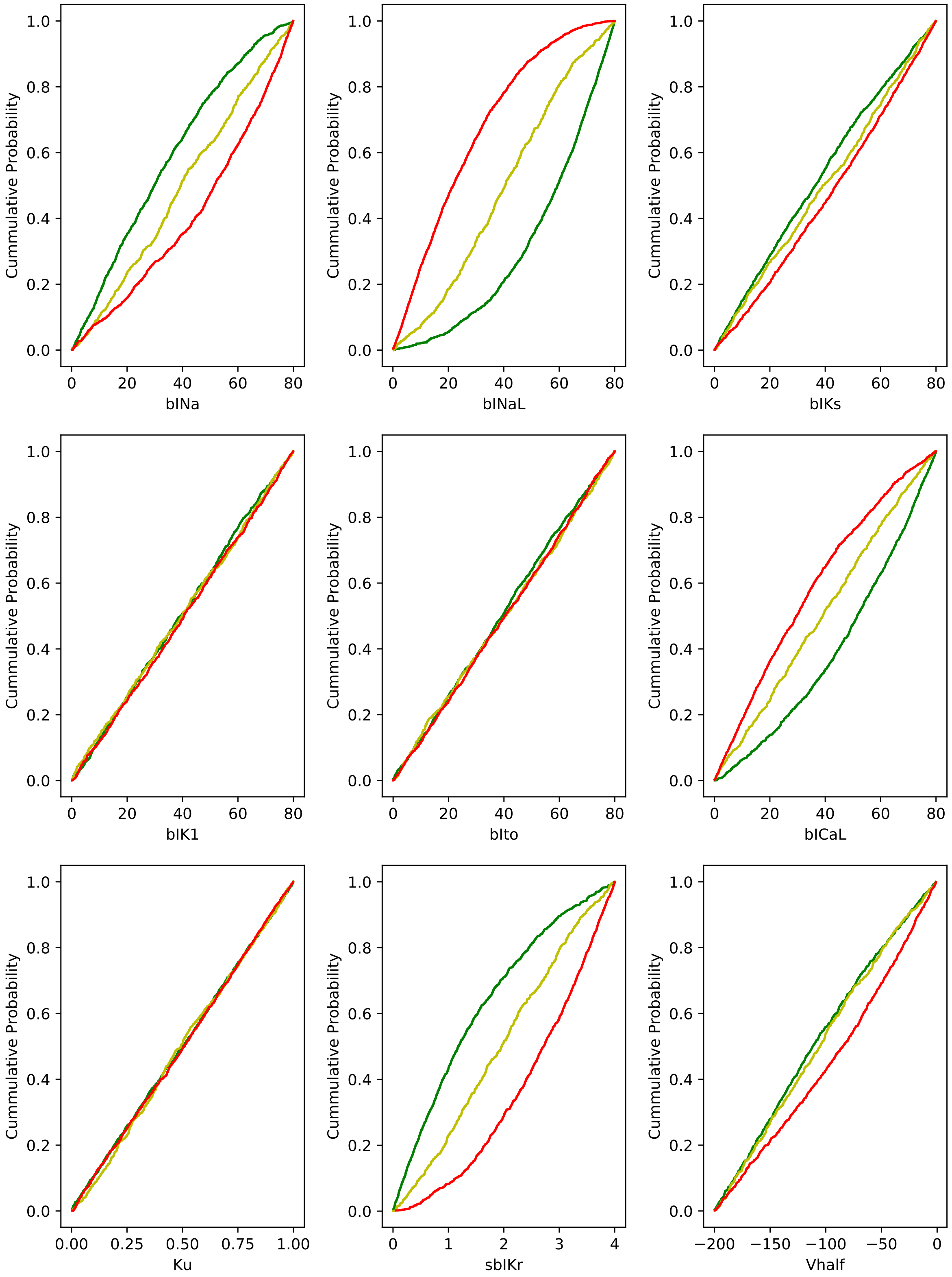

Figure S9. Ranking the most influential model parameters for separation of virtual drugs (generated using saltelli sampling over the parametric space) into low, intermediate and high risk groups based on the $qNet$ metric using Monte Carlo filtering analysis. Empirical CDF for each of the 9 input parameters conditional to the value of $qNet$. Green curve ($qNet \geq 70$), Yellow curve ($57 < qNet < 70$) and Red curve ($qNet \leq 57$.)

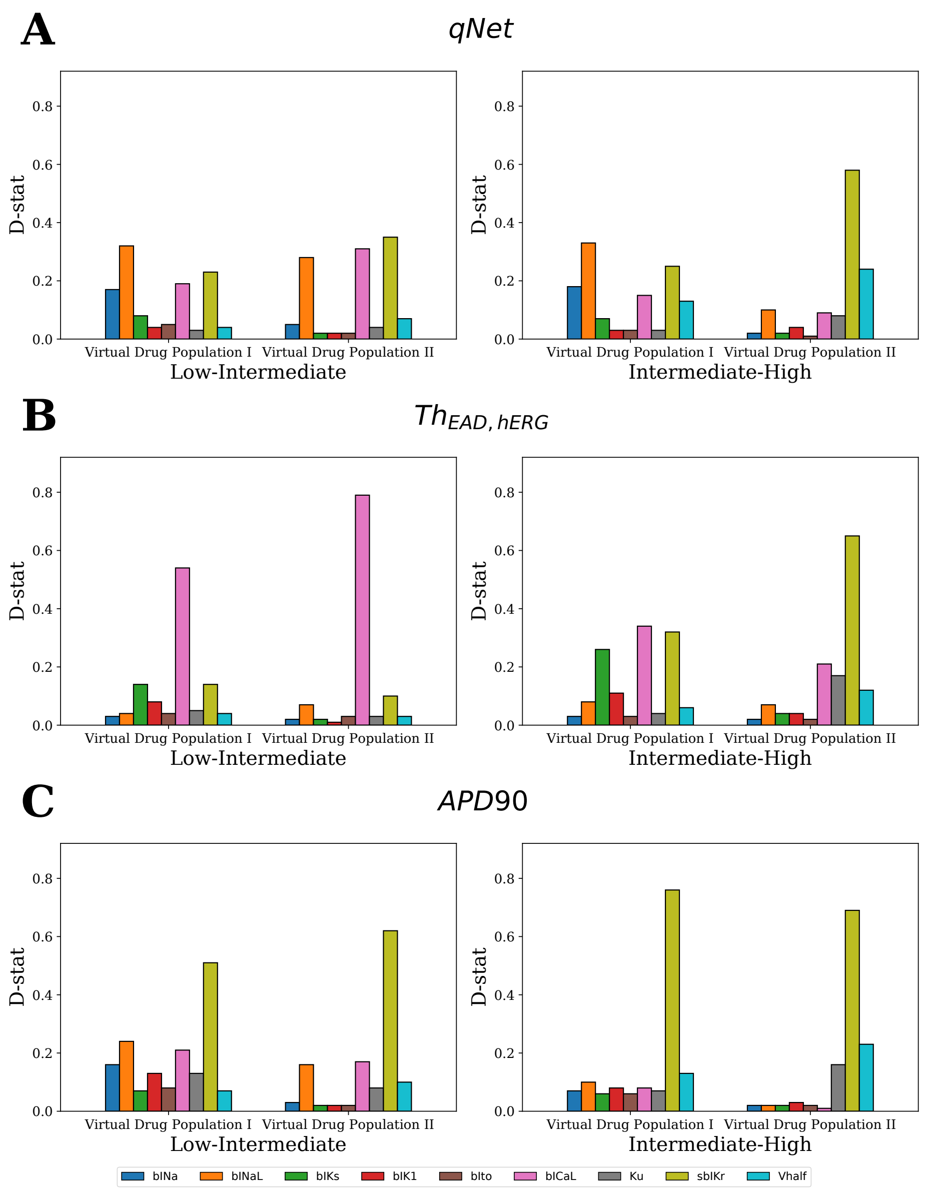

Figure S10. Ranking the most influential model parameters that allows separation of the virtual drugs into low, high and intermediate risk groups using Monte Carlo filtering analysis. Sensitivity measures obtained on separation of virtual drugs based on the **A:** $qNet$ metric, **B:** based on $Th_{EAD,hERG}$ metric, and **C:** based on APD90 metric. Virtual Drug Population I- 10000 virtual drugs generated using almost uniform sampling over the parametric space using saltelli sampling. Virtual Drug Population II- 10000 virtual drugs generated using prior distribution of each of the parameters for the 28 CiPA drugs.

1.5.3 Simple examples highlighting differences between Mote Carlo filtering versus logistic regression methods

Multivariate logistic regression estimates an hyperplane that provides best linear separation of the dichotomous outcome of interest (such as development of early after depolarizations). The regression coefficients of the estimated hyperplane can provide a good measure of the influence of the independent variables (parameters) on the occurrence of a dichotomous outcome Morotti and Grandi (2016). Here we illustrate few simple examples where the dichotomous outcome variable depends on only two independent parameters $P1$ and $P2$. In the case where the two groups are perfectly separable by a hyperplane, the logistic regression will accurately identify the separation surface and the regression coefficients will provide a good estimate of influence of the independent parameters (Figure S11). However, when the surface separating the dichotomous outcome of interest is not linear, the logistic regression method would not be able accurately identify the separation surface and the sensitivity measures determined by the regression coefficients would not provide good representation of the influence of the independent parameters depending on the complexity of the separation surface.

Monte Carlo filtering methods determines the influence of a individual independent parameter in isolation on separation of the binary outcome of interest Saltelli et al. (2008). The influence of the independent parameter is estimated by the maximum distance in the cumulative distribution function of the two subsets of the independent parameter categorized based on the dichotomous outcome.

On comparison of the logistic regression and Monte Carlo filtering methods using simple examples, we observe that for the case both the methods provide equivalent sensitivity estimates when the surface separating the dichotomous variable of interest if perfectly linear and the sample points are uniformly distributed over the entire region of interest. In the case, where a more complex surface separates the binary regions of interest, the two methods provide different sensitivity estimates (Figure S11). Monte Carlo filtering does not rely require the dichotomous variable to be perfectly separated by hyperplane and provide a more accurate measure even in presence of a complex separation surface. However, it should be noted that the Monte Carlo filtering provides the estimate of influence of individual parameter in isolation. Moreover, we observed that the sensitivity estimates provided by the two methods can be different on classification of non-uniformly sampled data points over the region of interest (see Figure S12). For example on separation of dichotomous variable of interest separated by the line $P1 = P2$, both the methods provide equivalent estimate in case of uniformly distributed data points in both the groups. However, on classification of data points based on some constraints and non-uniform distributions we observe that sensitivity measure can differ significantly. For the constrained case in example 2, Monte Carlo filtering reveal that the parameter $P2$ is more influential thant $P1$ whereas logistic regression will indicate that both the parameters are almost equally influential.

In addition we also calculated global sensitivity estimates using Mean Decrease Accuracy (MDA) method. We first approximate the separating surface by a metamodel (i.e., random forest classifier model for the examples). Then, we evaluated the accuracy of the metarepresentation upon random permutations of the values of a given feature. Losses in accuracy measured for each of the permutations provided us with global sensitivity estimates. Although not identical the sensitivity estimates obtained via MDA were similar to the estimates obtained via Monte Carlo filtering method.

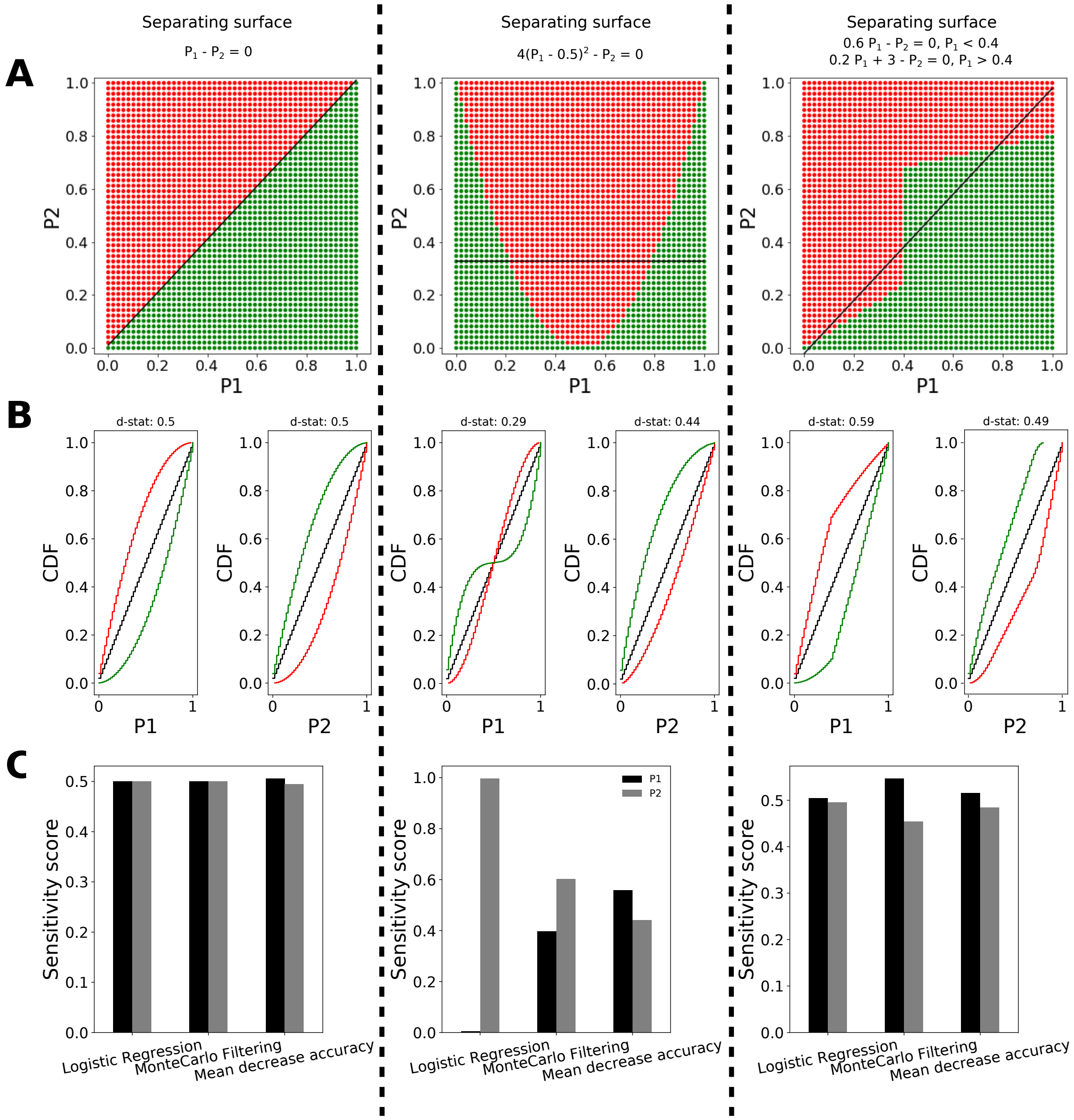

Figure S11. Comparison of sensitivity measures obtained via Monte Carlo filtering, logistic regression and MDA methods for hypothetical surfaces separating the behavioral and non-behavioral regions. **A:** Plots of three hypothetical separating surfaces. **B:** Empirical CDFs for both the input parameters $P1$ and $P2$. **C:** Bar plot comparing the obtained sensitivity indices with both the logistic regression and Monte Carlo filtering methods.

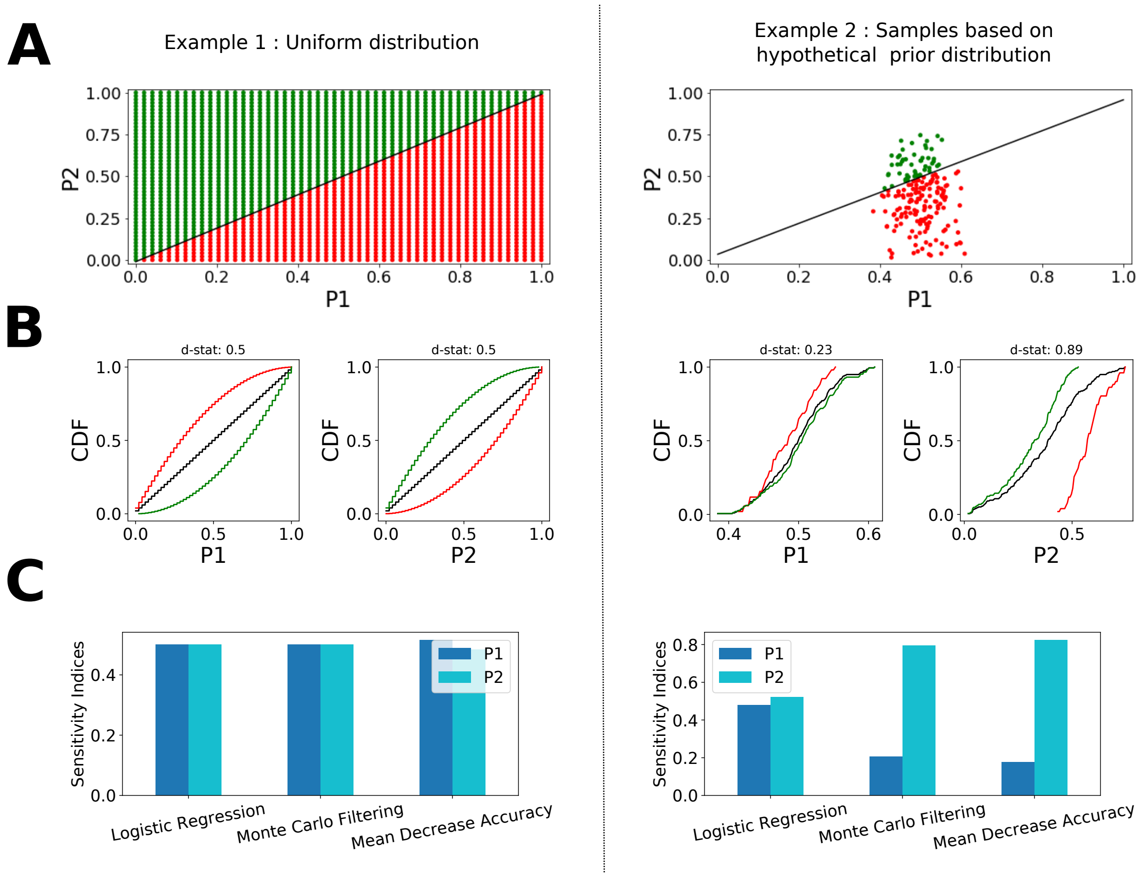

Figure S12. Comparison of sensitivity measures obtained via Monte Carlo Filtering, logistic regression and MDA methods for hypothetical surfaces separating the behavioral and non-behavioral regions. **A:** Plots of three hypothetical separating surfaces. **B:** Empirical CDFs for both the input parameters $P1$ and $P2$. **C:** Bar plot comparing the obtained sensitivity indices with both the logistic regression and Monte Carlo filtering methods.

REFERENCES

Crumb, W. J., Vicente, J., Johannesen, L., and Strauss, D. G. (2016). An Evaluation of 30 Clinical Drugs against the Comprehensive in Vitro Proarrhythmia Assay (CiPA) Proposed Ion Channel Panel. *Journal of Pharmacological and Toxicological Methods* 81, 251–262. doi:10.1016/j.vascn.2016.03.009

Li, Z., Dutta, S., Sheng, J., Tran, P. N., Wu, W., Chang, K., et al. (2017). Improving the In Silico Assessment of Proarrhythmia Risk by Combining hERG (Human Ether-à-Go-Go-Related Gene) Channel-Drug Binding Kinetics and Multichannel Pharmacology. *Circulation. Arrhythmia and Electrophysiology* 10, e004628. doi:10.1161/CIRCEP.116.004628

Li, Z., Ridder, B. J., Han, X., Wu, W. W., Sheng, J., Tran, P. N., et al. (2018). Assessment of an In Silico Mechanistic Model for Proarrhythmia Risk Prediction Under the CiPA Initiative. *Clinical Pharmacology & Therapeutics* 0. doi:10.1002/cpt.1184

Morotti, S. and Grandi, E. (2016). Logistic Regression Analysis of Populations of Electrophysiological Models to Assess Proarrythmic Risk. *MethodsX* 4, 25–34. doi:10.1016/j.mex.2016.12.002

Saltelli, A., Ratto, M., Andres, T., Campolongo, F., Cariboni, J., Gatelli, D., et al. (2008). *Global Sensitivity Analysis: The Primer* (Wiley)

Sobie, E. A. (2009). Parameter Sensitivity Analysis in Electrophysiological Models Using Multivariable Regression. *Biophysical Journal* 96, 1264–1274. doi:10.1016/j.bpj.2008.10.056
